# Correlates with Vaccine Protective Capacity and COVID-19 Disease Symptoms Identified by Serum Proteomics in Vaccinated Individuals

**DOI:** 10.3390/molecules27185933

**Published:** 2022-09-13

**Authors:** Margarita Villar, José Miguel Urra, Sara Artigas-Jerónimo, Lorena Mazuecos, Marinela Contreras, Rita Vaz-Rodrigues, Francisco J. Rodríguez-del-Río, Christian Gortázar, José de la Fuente

**Affiliations:** 1 SaBio, Instituto de Investigación en Recursos Cinegéticos IREC-CSIC-UCLM-JCCM, Ronda de Toledo 12, 13005 Ciudad Real, Spain; 2 Biochemistry Section, Faculty of Science and Chemical Technologies, and Regional Centre for Biomedical Research (CRIB), University of Castilla-La Mancha, 13071 Ciudad Real, Spain; 3 Immunology, Hospital General Universitario de Ciudad Real, 13005 Ciudad Real, Spain; 4 Medicine School, Universidad de Castilla la Mancha (UCLM), 13005 Ciudad Real, Spain; 5 Local Medical Service Horcajo de los Montes, 13110 Ciudad Real, Spain; 6 Department of Veterinary Pathobiology, Center for Veterinary Health Sciences, Oklahoma State University, Stillwater, OK 74078, USA

**Keywords:** COVID-19, proteomic, vaccine, immunology, biomarker

## Abstract

In the last two years, the coronavirus disease 19 (COVID-19) pandemic caused by severe acute respiratory syndrome coronavirus 2 (SARS-CoV-2) has been a scientific and social challenge worldwide. Vaccines have been the most effective intervention for reducing virus transmission and disease severity. However, genetic virus variants are still circulating among vaccinated individuals with different disease symptomatology. Understanding the protective- or disease-associated mechanisms in vaccinated individuals is relevant to advances in vaccine development and implementation. To address this objective, serum-protein profiles were characterized by quantitative proteomics and data-analysis algorithms in four cohorts of uninfected and SARS-CoV-2-infected vaccinated individuals with asymptomatic, non-severe, and severe disease symptomatology. The results show that immunoglobulins were the most overrepresented proteins in infected cohorts when compared to PCR-negative individuals. The immunoglobulin profile varied between different infected cohorts and correlated with protective- or disease-associated capacity. Overrepresented immunoglobulins in PCR-positive individuals correlated with protective response against SARS-CoV-2, other viruses, and thrombosis in asymptomatic cases. In non-severe cases, correlates of protection against SARS-CoV-2 and HBV together with risk of myasthenia gravis and allergy and autoantibodies were observed. Patients with severe symptoms presented risk for allergy, chronic idiopathic thrombocytopenic purpura, and autoantibodies. The analysis of underrepresented immunoglobulins in PCR-positive compared to PCR-negative individuals identified vaccine-induced protective epitopes in various coronavirus proteins, including the spike receptor-binding domain RBD. Non-immunoglobulin proteins were associated with COVID-19 symptoms and biological processes. These results evidence host-associated differences in response to vaccination and the possibility of improving vaccine efficacy against SARS-CoV-2.

## 1. Introduction

Millions of deaths have been reported worldwide associated with coronavirus disease 19 (COVID-19), a pandemic caused by severe acute respiratory syndrome coronavirus 2 (SARS-CoV-2) (https://www.google.com/search?client=safari&rls=en&q=COVID-19+worldwide+cases&ie=UTF-8&oe=UTF-8#colocmid=/m/02j71&coasync=0).

**Figure 1:**
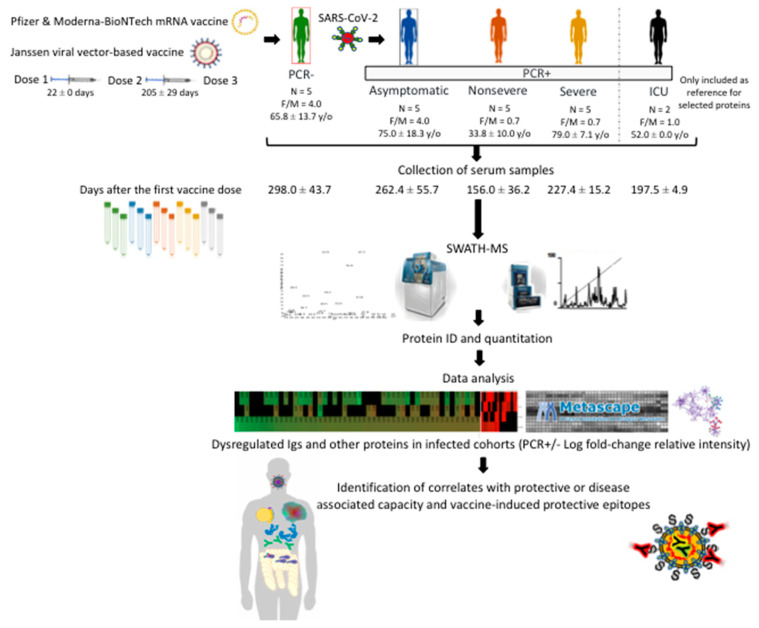
Experimental design and rationale. The experimental design used in our study was based on sera collected from vaccinated individuals (mostly with Pfizer and Moderna-BioNTech) and SARS-CoV-2 PCR-negative (PCR–) or infected PCR-positive (PCR+) and with asymptomatic, non-severe, and severe COVID-19 symptomatology (Table 1). Two PCR+ cases with severe symptoms and in the hospital intensive-care unit (ICU) were included only as reference for selected proteins. Sera were collected between 156 and 298 days after first vaccine-dose administration and subjected to SWATH-MS quantitative proteomics to characterize serum-protein profiles in different cohorts. The proteomics results were then translated into the identification of correlates with protective- or disease-associated capacity and vaccine-induced protective epitopes.

Furthermore, the global number of deaths caused by COVID-19 may be up to four times this figure [1]. Vaccines using different platforms have been developed as the most safe and effective intervention for reducing SARS-CoV-2-virus transmission and disease severity [2,3]. However, genetic variants of the coronavirus are still circulating among vaccinated individuals with different disease symptomatology [4,5]. Understanding the protective- or disease host-associated mechanisms in vaccinated individuals is relevant to advances in vaccine development and implementation [6].

To address this challenge, understanding the protective- or disease-associated mechanisms in vaccinated individuals is relevant to advances in vaccine development and implementation. Previous proteomics studies, e.g., [7,8], have addressed response to infection and vaccination, but our study addressed for the first time the immune response to COVID-19 vaccination in uninfected and SARS-CoV-2-infected individuals with asymptomatic, non-severe, and severe disease symptomatology. In this study, serum-protein profiles were characterized by previously validated quantitative proteomics [7] in four cohorts of uninfected and SARS-CoV-2-infected vaccinated individuals with asymptomatic, non-severe, and severe disease symptomatology. The results evidence host-associated differences in response to vaccination and the possibility of advances in vaccine development and implementation against SARS-CoV-2.

## 2. Materials and Methods

### 2.1. Study Design with Serum Samples from Different Cohorts

The study design is described in Figure 1. A retrospective case-control study was conducted in patients suffering from COVID-19 and healthy controls sampled at the University General Hospital of Ciudad Real (HGUCR), Spain [8]. Individuals were confirmed as SARS-CoV-2-infected by reverse transcriptase-polymerase chain reaction (RT-PCR) and sampled between November and December 2021 (Table 1). In this study with individuals vaccinated against COVID-19, vaccine administration, clinical symptoms, and laboratory determinations associated with COVID-19 were obtained from patients’ medical records to create cohorts of PCR– and PCR+ asymptomatic, non-severe, and severe individuals (Table 1). Patient symptoms can be found in Table 1. Blood samples were drawn in a vacutainer tube without anticoagulant. The tube remained at rest for 15–30 min at room temperature (RT) for clotting. Subsequently, the tube was centrifuged at 1500× *g* for 10 min at RT to remove the clot and obtain serum. Serum samples were heat-inactivated for 30 min at 56 °C and conserved at −20 °C until used for analysis. The use of samples and individuals’ data was approved by the Ethical and Scientific Committees (University Hospital of Ciudad Real C-352 and SESCAM C-73).

### 2.2. Serum Proteomics

The methodology and algorithms for serum proteomics were as previously described [8]. Serum samples from PCR– controls and PCR+ COVID-19 asymptomatic, non-severe, and severe individuals (n = 5 each) were individually analyzed. Two PCR+ cases with severe symptoms and in the hospital ICU were included only as reference for the selected proteins. Protein concentration in samples was determined using the BCA Protein Assay with BSA (Sigma-Aldrich) as standard. Protein-serum samples (100 µg per sample) were trypsin digested using the FASP Protein Digestion Kit (Expedeon Ltd., UK) and sequencing-grade trypsin (Promega, Madison, WI, USA) following the manufacturer’s recommendations. The resulting tryptic peptides were desalted onto OMIX Pipette tips C18 (Agilent Technologies, Santa Clara, CA, USA), dried down, and stored at −20 °C until mass-spectrometry analysis. The desalted protein digests were resuspended in 2% acetonitrile, 5% acetic acid in water, and analyzed by reverse-phase liquid chromatography coupled with mass spectrometry (RP-LC-MS/MS) using an Ekspert^TM^ nanoLC 415 system coupled online with a 6600 TripleTOF^®^ mass spectrometer (AB SCIEX; Framingham, MA, USA) through Information-Dependent Acquisition (IDA) followed by Sequential Windowed data-independent Acquisition of the Total High-resolution Mass Spectra (SWATH-MS). The peptides were concentrated in a 0.1 × 20 mm C18 RP precolumn (Thermo Fisher Scientific, Waltham, MA, USA) with a flow rate of 2 µL/min for 10 min in solvent A. Then, peptides were separated in a 0.075 × 250 mm C18 RP column (New Objective, Woburn, MA, USA) with a flow rate of 300 nl/min. Elution was carried out in a 120 min gradient from 5% B to 30% B followed by a 15 min gradient from 30% B to 60% B (Solvent A: 0.1% formic acid in water, solvent B: 0.1% formic acid in acetonitrile) and directly injected into the mass spectrometer for analysis. For IDA experiments, the mass spectrometer was set to scanning full spectra from 350 *m*/*z* to 1400 *m*/*z* (250 ms accumulation time), followed by up to 50 MS/MS scans (100–1500 *m*/*z*). Candidate ions with a charge state between +2 and +5 and counts per second above a minimum threshold of 100 were isolated for fragmentation. One MS/MS spectrum was collected for 100 ms before adding those precursor ions to the exclusion list for 15 s (mass spectrometer operated by Analyst^®^ TF 1.6, ABSciex^®^). Dynamic background subtraction was turned off. Data were acquired in high-sensitivity mode with rolling collision energy on and a collision energy spread of 5. An equal amount of the five samples for each experimental group joined together as a representative mixed sample of each of the 4 experimental groups, which were used for the generation of the reference spectral-ion library as part of SWATH-MS analysis. A total amount of 4 µg protein digests for each mixed sample was injected. For SWATH quantitative analysis, 50 independent samples (2 technical replicates from each of the 5 biological replicates for each of the 4 experimental groups and 5 technical replicates from each of the 2 biological replicates in the case of ICU samples) (6 μg each) were subjected to the cyclic data-independent acquisition (DIA) of mass spectra using the SWATH variable-window calculator (V 1.0, AB SCIEX) and the SWATH acquisition-method editor (AB SCIEX), similar to established methods [8]. A set of 50 overlapping windows was constructed (containing 1 *m*/*z* for the window overlap), covering the precursor mass range of 400–1250 *m*/*z*. For these experiments, a 50 ms survey scan (350–1400 *m*/*z*) was acquired at the beginning of each cycle, and SWATH-MS/MS spectra were collected from 100–1500 *m*/*z* for 70 ms in high-sensitivity mode, resulting in a cycle time of 3.6 s. Collision energy for each window was determined according to the calculation for a charge +2 ion centered upon the window with a collision-energy spread of 15. To create a spectral library of all the detectable peptides in the samples, the IDA MS raw files were combined and subjected to database searches in unison using ProteinPilot software v. 5.0.1 (AB SCIEX) with the Paragon algorithm. Spectra identification was performed by searching against the Uniprot human-proteome database (79,038 entries in January 2022) with the following parameters: iodoacetamide cysteine alkylation, trypsin digestion, identification focus on biological modification, and thorough ID as search effort. The detected protein threshold was set at 0.05. To assess the quality of identifications, an independent False Discovery Rate (FDR) analysis with the target-decoy approach provided by Protein Pilot^TM^ was performed. Positive identifications were considered when identified proteins reached a 1% global FDR. The mass-spectrometry proteomics data were deposited in the Proteome Xchange Consortium via the PRIDE partner repository with the dataset identifier PXD031969 and 10.6019/PXD031969.

### 2.3. Quality Control of Proteomics Data

The quality of the proteomics data was controlled at multiple levels. First, a rat-ileum digest was used for the evaluation of instrument performance. Buffer A samples were run as blanks every two injections to prevent carryover. Two technical replicates were injected for each sample. For validation of serum-proteomics data, protein representation for previously identified selected biomarkers for COVID-19 and proteomics studies were used to show correlation with disease severity. An enrichment analysis was conducted using the Coronascape COVID database (https://metascape.org/COVID; [8]) to identify proteins found in our study as differentially represented in response to COVID-19 and reported in previous COVID-19 omics datasets. 

### 2.4. Data Analysis

For SWATH processing, up to 10 peptides with 7 transitions per protein were automatically selected by the SWATH Acquisition MicroApp 2.0 in the PeakView 2.2 software with the following parameters: 15 ppm ion library tolerance, 5 min XIC extraction window, 0.01 Da XIC width, and considering only peptides with at least 99% confidence and excluding those that were shared or contained modifications. However, to ensure reliable quantitation, only proteins with 3 or more peptides available for quantitation were selected for XIC peak-area extraction and exported for analysis in the MarkerView 1.3 software (AB SCIEX). Global normalization according to the total area sums of all detected proteins in the samples was conducted (Supplementary Materials Data File S1). The Student’s *t*-test (*p* < 0.05) was used to perform two-sample comparisons between the averaged area sums of all the transitions derived for each protein across the 10 replicate runs for each group under comparison to identify proteins that were significantly represented between groups (Supplementary Materials Data File S1). Protein representation was compared between groups by One-way ANOVA test followed by post-hoc Bonferroni and Holm multiple comparisons tests (*p* < 0.05; https://astatsa.com/OneWay_Anova_with_TukeyHSD/), and relative intensity was compared between PCR- and PCR+ cohorts by Welch’s unpaired *t*-test (*p* < 0.05; https://www.graphpad.com/quickcalcs/ttest1/?Format=C) [8]. Data were separately analyzed for overrepresented and underrepresented proteins using the Metascape gene annotation and analysis resource (https://metascape.org/gp/index.html#/main/step1). The analytical algorithm developed using Protein BLAST sequence alignment against non-redundant protein database (nr) using compositional matrix adjustment (https://blast.ncbi.nlm.nih.gov/Blast.cgi?PROGRAM = blastp&PAGE_TYPE = BlastSearch&LINK_LOC = blasthome) and Paratome (http://www.ofranlab.org) was used for the identification of antigen-binding regions and vaccine-induced antibody-protective epitopes and correlates of identified proteins with protective- or disease-associated capacity (Supplementary Materials Data Files S2 and S4).

### 2.5. Antibody Neutralization Test

Antibody titers specific for the neutralization of SARS-CoV-2 virus were determined with a cPass SARS-CoV-2 neutralization antibody-detection kit (Genscript, Piscataway, NJ, USA) following the manufacturer′s instructions. Briefly, 100  µL of the positive and negative controls and serum samples at 1:100 dilution and previously incubated with HRP conjugated RBD during 30 min at 37 °C were added to the 96-microwell plate coated with RBD-SARS-CoV-2 protein and incubated for 15 min at 37 °C. After washing four times with 260  µL/well of wash buffer, 100  µL/well of chromogen-substrate solution were added and incubated for 15  min at RT. Finally, the colorimetric reaction was stopped with 50  µL/well of stop solution and the absorbance was measured in a spectrophotometer (Thermo Fisher Scientific) at O.D. 450  nm. Results were evaluated by calculating the ratio between the O.D. of the sample and the O.D. of the calibrator using the following formula: Inhibition = (1 − O.D. value of sample/O.D. value of negative control) × 100.

### 2.6. Antibody Levels against HBV and Zika Virus

Individual sera from all cohorts included in the study were characterized for antibody levels against HBV and Zika virus using pathogen-specific ELISA tests. The ELISA kits are designed for the detection of antibodies to hepatitis B-virus surface antigen: Hepatitis B surface antigen Ab ELISA kit (AB-KA0287; Biogen Científica, Madrid, Spain) or anti-Zika Virus Non-structural Protein, Anti-Zika virus IgG ELISA kit (ab221844; Abcam, Cambridge, UK) in human serum. Antibody levels were compared between PCR– and PCR+ cohorts by Chi-squared test (*p* < 0.05). A Spearman’s Rho (*rs*) correlation analysis was conducted between virus cross-reactive Ig levels and proteomic-protein relative intensity (*p* < 0.05; https://www.socscistatistics.com/tests/spearman/default2.aspx).

### 2.7. Human Autoantibody General Survey Microarray

Reactive autoantibodies were characterized with individual sera from all cohorts using the Human Autoantigens General Survey Antigen Microarray (GeneCopoeia’s OmicsArray; Rockville, MD, USA), a protein microarray enabling powerful detection of autoantibodies associated with many diseases, including rheumatoid arthritis, muscular dystrophy, systemic lupus erythematosus, and type-1 diabetes. The array carries 120 superior-quality purified proteins spotted onto nitrocellulose filters, which are adhered to glass slides. Antigens known to be associated with specific autoimmune diseases are chosen based on a thorough review of peer-reviewed publications. Data interpretation: NSI, net signal intensity, averaged fluorescent-signal intensity for each antigen subtracted by local background and negative control signal; NSI-nor, NSI normalized to internal Ig controls; SBR, averaged signal-to-background ratio of each antigen; SBR-nor, SBR normalized to internal Ig controls; SNR, signal-to-noise ratio that represents the significance of the signal above the background (SNR ≥ 3 means the signal is significantly higher than the background). Additional information is in Supplementary Materials Data File S3.

## 3. Results and Discussion

### 3.1. Characterization of Immunoglobulin Protein Profiles and Correlation with Protective- or Disease-Associated Capacity

**Figure 2:**
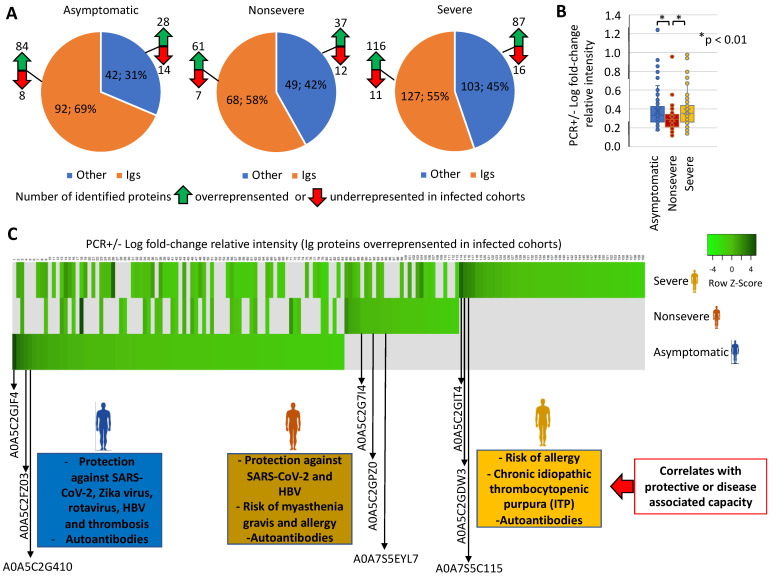
Serum-protein profiles and correlation of Ig overrepresented in vaccinated infected cohorts with protective- or disease-associated capacity. (**A**) Number of identified overrepresented or underrepresented Ig and non-Ig proteins in infected asymptomatic, non-severe, and severe cohorts when compared to PCR– individuals. (**B**) Change in Ig-protein levels in PCR+ cohorts. PCR+/− Log fold-change relative intensity was compared between groups by One-way ANOVA test followed by post-hoc Bonferroni and Holm multiple comparisons tests (*p* < 0.05). (**C**) Heatmap of PCR+/− Log fold-change relative intensity (Z-scored original value) for Ig proteins overrepresented in infected cohorts. Correlates with protective- or disease-associated capacity are shown for Igs highly overrepresented in PCR+ individuals.

The experimental design used in our study was based on sera collected from SARS-CoV-2 PCR-negative (PCR–) or infected PCR-positive (PCR+) vaccinated individuals (mostly with Pfizer and Moderna-BioNTech) and with asymptomatic, non-severe, and severe COVID-19 symptomatology (Figure 1, Table 1). Two PCR+ cases with severe symptoms and in the hospital intensive-care unit (ICU) were included only as reference for selected proteins. Sera were collected in November–December 2021 before the appearance of the SARS-CoV-2 Omicron variant in Spain, and thus most PCR+ cases were probably of the Delta virus variant (Table 1). As expected, 91% of the individuals’ serum samples (20 out of 22 except for samples C5 and S1; Table 1) showed neutralization antibodies against SARS-CoV-2. Nevertheless, the characterization of antibody- and non-antibody-mediated immune response to vaccination is important to understanding the response to COVID-19 vaccines. Proteomics analysis identified significantly dysregulated proteins in vaccinated and infected cohorts (asymptomatic, n = 134; non-severe, n = 117; severe, n = 230) when compared to vaccinated PCR– individuals (Figure 2A, Supplementary Materials Data File S1). More than 55% of the dysregulated proteins identified in infected cohorts when compared to PCR– individuals were immunoglobulins (Igs), mostly overrepresented in asymptomatic and severely infected cohorts (Figure 2A,B). This result is associated with response to vaccination, as supported by the finding using a similar serum-proteomics approach in healthy, unvaccinated individuals and with different COVID-19 symptomatology, in whom only 32% (60/189) of the dysregulated proteins were Igs [8]. 

An analytical workflow was developed to characterize selected Ig proteins identified as significantly dysregulated in vaccinated and infected cohorts when compared to vaccinated PCR– individuals (Supplementary Materials Data File S2). The use of the Paratome web server (http://www.ofranlab.org) allowed for the identification of antigen-binding regions in identified Ig light- or heavy-chain variable regions, including but not limited to complementarity-determining regions (CDRs) [9]. As has been discussed, heavy-chain complementarity-determining region 3 (HCDR3) is necessary, but insufficient for specific antibody binding [10].

**Figure 3:**
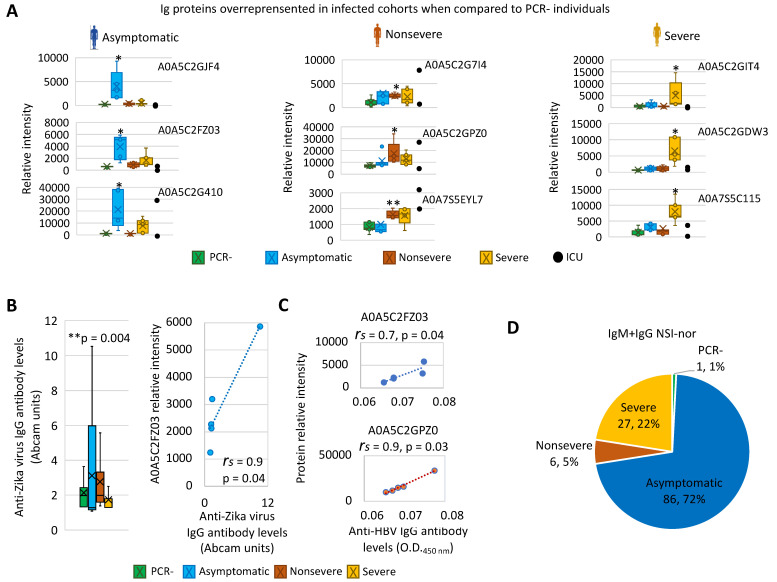
Validation of serum Ig proteins overrepresented in vaccinated infected individuals. (**A**) Changes in Ig-protein mass-spectra relative intensity for selected overrepresented Ig in individual samples in all PCR– and PCR+ cohorts. Relative intensity was compared between PCR– and PCR+ cohorts by Welch’s unpaired t-test (* *p* < 0.05, ** *p* < 0.005; n = 5 biological replicates). Samples from individuals in the ICU were only included as reference for selected proteins. (**B**) Independent analysis of predicted biomarker A0A5C2FZ03 for anti-Zika virus Ig levels. Antibody levels were compared between PCR– and PCR+ cohorts by Chi-squared test (*p* = 0.004 for asymptomatic cohort). A Spearman’s Rho correlation analysis was conducted between virus cross-reactive Ig levels and proteomics-protein relative intensity (*p* < 0.05). (**C**) Independent analysis of predicted biomarkers A0A5C2FZ03 and A0A5C2GPZ0 for anti-HBV Ig levels in asymptomatic and non-severe cohorts, respectively. A Spearman’s Rho correlation analysis was conducted between HBV cross-reactive Ig levels and proteomics-protein relative intensity (*p* < 0.05). (**D**) A human-autoantibody general survey identified proteins reactive to IgM and IgG autoantibodies with significant NSI-nor values in PCR– and PCR+ cohorts. Proteins were distributed based on the highest NSI-nor average IgM+IgG value.

Focusing on Igs highly overrepresented in PCR+ individuals, the results showed differences between infected cohorts (Figure 2C, Supplementary Materials Data File S2). In vaccinated and infected asymptomatic cases, predictive models associated the Igs with protection against SARS-CoV-2, Zika virus, rotavirus, Hepatitis B virus (HBV), and thrombosis. However, in cases with COVID-19 symptoms, Igs associated with protection against SARS-CoV-2 and HBV were only identified in non-severe cases, whereas Igs associated with autoantibodies and risk of allergic reactions and diseases such as myasthenia gravis and chronic idiopathic thrombocytopenic purpura (ITP) were identified only in non-severe and severe patients (Figure 2C, Supplementary Materials Data File S2). Additionally, autoantibodies were identified in all PCR+ cohorts. The analysis of mass-spectra relative intensity for selected overrepresented Ig proteins corroborated in individual samples the cohort-dependent results (Figure 3A). Then, an independent analysis of predicted biomarkers was used to validate these results for Zika virus, HBV, and a human-autoantibody general survey (Figure 3B–D). 

In agreement with serum-protein profiles and correlation of Igs overrepresented in vaccinated infected cohorts with protective-associated capacity (Figure 2C and Figure 3A), the results showed that the anti-Zika-virus IgG levels were significantly higher in the asymptomatic cohort only (Figure 3B). Furthermore, a significant positive correlation between Zika-virus cross-reactive Ig levels and relative intensity of associated A0A5C2FZ03 protein was obtained (Figure 3B, Supplementary Materials Data File S2). For HBV, the sensitivity of the surface-antigen ELISA (positive values > 0.034) did not allow for identification of positive individuals in PCR+ cohorts and allowed identification of only one individual in the PCR– cohort (C2 in Table 1, value = 0.079). Nevertheless, as predicted for serum-protein profiles (Figure 2C and Figure 3A), a significant positive correlation was obtained between mean HBV ELISA O.D. 450 nm values and relative intensity of associated proteins A0A5C2FZ03 and A0A5C2GPZ0 only in asymptomatic and non-severe individuals, respectively (Figure 3C, Supplementary Materials Data File S2). 

**Figure 4:**
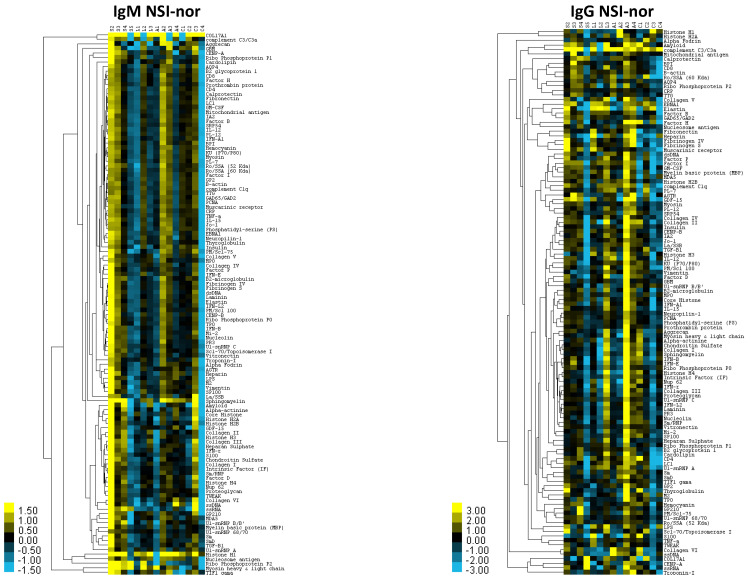
Heatmap of IgM and IgG autoantibodies overrepresented in vaccinated infected cohorts. According to the NSI-nor value, log2 (NSI+1) is calculated, the data are normalized, and a heat map is generated. The antigens are clustered according to Euclidean distance. Additional information is in Supplementary Materials Data File S3. Abbreviations: S, severe; L, non-severe: A, asymptomatic; C, PCR– (Table 1).

Autoantibodies in COVID-19 patients have been recently correlated with increased antiviral humoral and inflammatory immune responses [11]. In our study regarding autoantibodies, the survey identified 120 target proteins with significant signal-to-noise ratio (SNR ≥ 3; Figure 3D, Supplementary Materials Data File S3). The net signal intensity for each antigen subtracted by the local background and negative control signal and normalized to internal Ig controls (NSI-nor; Supplementary Materials Data File S3) showed a distribution by different cohorts with the highest NSI-nor average IgM+IgG value (IgM+IgG NSI-nor; Figure 3D). In accordance with serum-protein profiles, autoantibodies were identified mostly in PCR+ cohorts with only one protein (Histone H1), with the highest IgM+IgG NSI-nor value in PCR– individuals (Figure 3D and Figure 4, Supplementary Materials Data File S3). As predicted by our analysis (Supplementary Materials Data File S2), most of the identified proteins reactive to autoantibodies were involved in the regulation of the immune system and/or associated with different diseases (Figure 4, Supplementary Materials Data File S3). For example, nuclear pore-membrane glycoprotein GP210 identified with the highest IgM+IgG NSI-nor value in the severe cohort is a prognostic marker in patients with primary biliary cirrhosis [12]. Another protein with significant signal-to-noise ratio in all PCR– and PCR+ cohorts (SNR ≥ 4.5) and the highest IgM+IgG NSI-nor in severe patients (SNR > 12) was the Type-1 angiotensin II receptor (AGTR; Supplementary Materials Data File S3), which during SARS-CoV-2 infection can recognize and internalize the soluble angiotensin-converting enzyme 2 (ACE2)–coronavirus spike protein complex through dynamin 2-dependent endocytosis [13]. In accordance with our results, these autoantibodies are associated with an unfavorable COVID-19 disease course [14].

**Figure 5:**
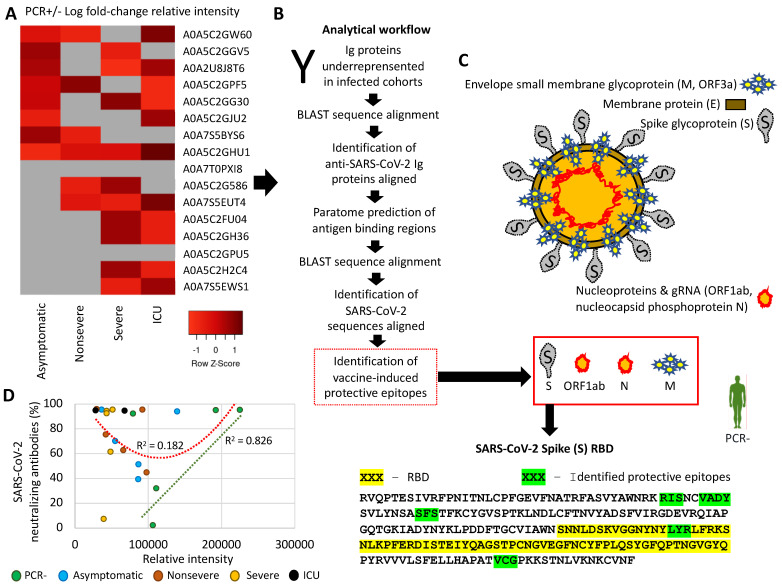
Serum-protein profiles of Ig underrepresented in vaccinated infected cohorts and identification of vaccine-induced protective epitopes. (**A**) Heatmap of PCR+/− Log fold-change relative intensity (Z-scored original value) for Ig proteins underrepresented in infected cohorts. (**B**) Analytical workflow developed for the dentification of vaccine-induced protective epitopes. (**C**) Identification of SARS-CoV-2 proteins with predicted reactive epitopes to Ig underrepresented in infected cohorts and thus overrepresented in PCR– individuals. All methods and results are disclosed in Supplementary Materials Data File S2. (**D**) Correlation analysis between SARS-CoV-2-neutralizing antibodies (Table 1) and mass-spectra relative intensity of identified Igs with vaccine-induced protective epitopes (Supplementary Materials Data Files S1 and S2).

The Igs identified as underrepresented in infected cohorts and thus with higher relative levels in PCR– individuals (Figure 5A, Supplementary Materials Data File S2) were used for the identification of vaccine-induced protective epitopes using a designed analytical workflow (Figure 5B, Supplementary Materials Data File S2). The results showed that the protective epitopes were not only identified in the SARS-CoV-2 spike (S) receptor-binding domain (RBD) associated with vaccine-protective capacity [15], but also in other virus proteins such as envelope small-membrane glycoprotein M (ORF3a), membrane-protein E, and nucleocapsid phosphoprotein N (ORF1ab) (Figure 5C, Supplementary Materials Data File S2). A correlation analysis was conducted between SARS-CoV-2-neutralizing antibodies (Table 1) and mass-spectra relative intensity of identified Igs with vaccine-induced protective epitopes (Supplementary Materials Data Files S1 and S2). The results showed no significant correlation for all cohorts together (R^2^ = 0.182) but revealed a positive correlation in PCR– individuals in whom these proteins were overrepresented (R^2^ = 0.826), thus providing support to the predicted protective epitopes in response to vaccination (Figure 5D). 

These results further advance our knowledge on the antibody response in vaccinated uninfected (fully protected) and vaccinated SARS-CoV-2-infected (partially protected) individuals associated with host factors such as age, comorbidities, and coronavirus infection [16,17,18,19]. Whereas vaccinated PCR– individuals developed a protective response mediated by Igs against multiple SARS-CoV-2 proteins to prevent infection, PCR+ individuals showed overrepresented Ig profiles associated with COVID-19 symptomatology with protective Igs to control virus infection and thrombosis in asymptomatic cases and limited or no protective response against SARS-CoV-2 with Ig-associated risk of allergy and other diseases in non-severe and severe patients.

### 3.2. Characterization of Non-Ig Protein Profiles and Correlation with COVID-19

**Figure 6:**
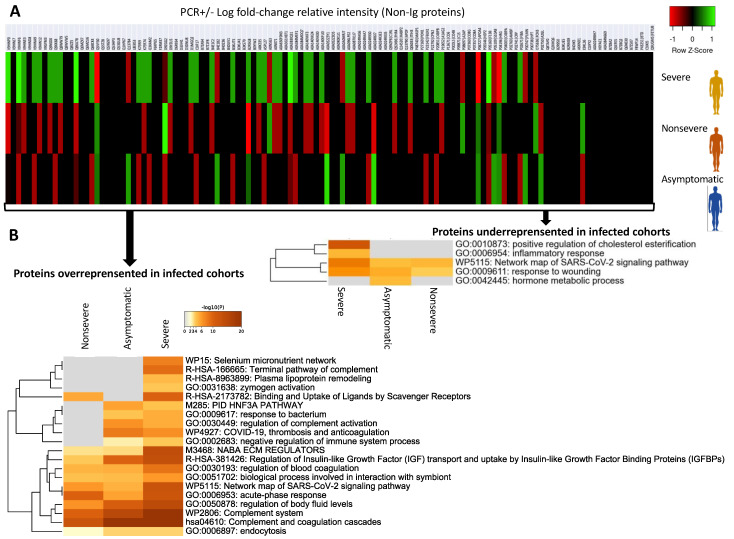
Multiple differential representation and enrichment ontology clusters for non-Ig proteins. (**A**) Heatmap of PCR+/− Log fold-change relative intensity (Z-scored original value). (**B**) Enriched GO/KEGG ontology clusters for proteins over- and underrepresented in infected cohorts when compared to PCR– cases. Accumulative hypergeometric *p*-values and enrichment factors were calculated and used for filtering. Remaining significant terms were then hierarchically clustered into a tree based on Kappa-statistical similarities among their gene memberships. Then, the 0.3 kappa score was applied as the threshold to cast the tree into term clusters. The term with the best *p*-value within each cluster was selected as its representative term and displayed in a dendrogram.

**Figure 7:**
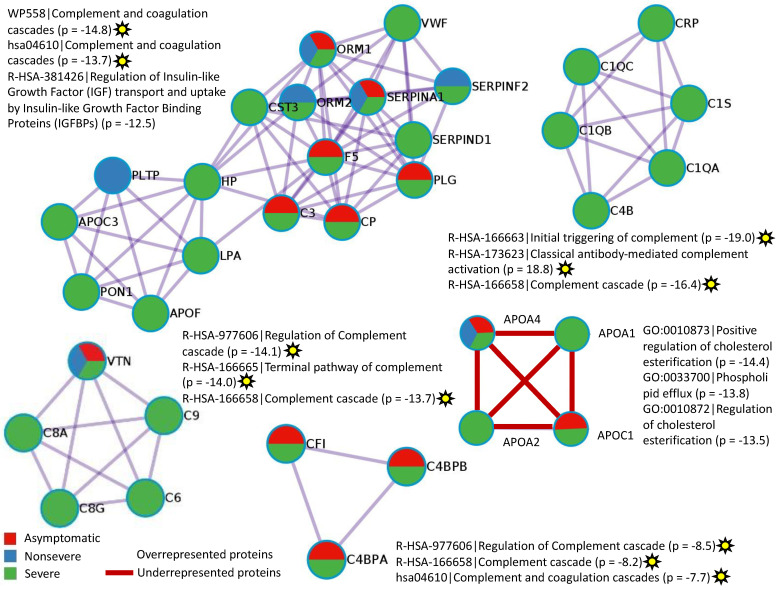
Protein–protein-interaction networks and components for non-Ig proteins over- and underrepresented in infected cohorts when compared to PCR– cases. The MCODE algorithm was applied to networks to identify neighborhoods where proteins are densely connected. GO enrichment analysis was applied to the original protein–protein-interaction network and its MCODE network components to extract their “biological meanings,” where the top three best *p*-value terms were retained. GOs with proteins involved in the regulation of complement and coagulation cascades and antibody-mediated complement activation are identified with yellow stars.

As reported in previous proteomics studies [7,8], identified dysregulated non-Ig proteins and biological processes in vaccinated infected PCR+ cohorts when compared to vaccinated PCR– individuals were associated with SARS-CoV-2 infection and COVID-19 (Figure 6A,B and Figure 7, Supplementary Materials Data Files S1 and S4). As expected, PCR+/− Log fold-change relative intensity was higher in individuals with severe symptoms (Figure 6A,B). Accordingly, protein–protein-interaction networks and components for non-Ig proteins over and underrepresented in infected cohorts showed a higher representation in the severe cohort when compared to PCR– cases (Figure 7). Gene-ontology (GO) categories with overrepresented proteins involved in the regulation of complement and coagulation cascades and antibody-mediated complement activation were the most represented in protein–protein interactions (Figure 7; identified with yellow stars). Hyperactivation of the complement and coagulation systems are associated with the clinical syndrome of COVID-19 [20]. 

The other GO identified in the protein–protein-interaction network of overrepresented proteins was the insulin-like growth factor (IGF) pathway as seen in Figure 7. Although an association has been proposed between low IGF1 levels and poor outcome in patients with COVID-19 [21], an epidemiological study provided evidence that higher IGF-1 concentrations are associated with a lower risk of COVID-19 mortality [22]. The results of our study suggested that activation of the IGF pathway may occur in response to vaccination by regulating immune-cell homeostasis to reduce risk for COVID-19 mortality [23].

Another finding of our study was related to severe-cohort overrepresented proteins in the biological process involved in interaction with symbiont (GO:0051702) (Figure 6B, Supplementary Materials Data File S4). One of the proteins identified in this biological process, Apolipoprotein E isoform 1 (APOE1; A0A0S2Z3D5), was overrepresented in severe (Log fold-change = 0.157) and UCI (Log fold-change = 0.081) patients (Supplementary Materials Data File S1). The expression of ApoE proteins, including APOE1, is critical for the assembly of infectious Hepatitis C virus (HCV) in a strain-specific and cell-type dependent manner [24]. Related to COVID-19, higher disease risk has been associated with *apoE4* genetic variants [25], but this is the first possible implication of ApoE1 in this process. Therefore, APOE1-protein levels and genetic variants may be a biomarker associated with disease severity in vaccinated and SARS-CoV-2-infected individuals.

As in recent studies [26], the interacting underrepresented proteins in vaccinated and infected cohorts were apolipoproteins APOA1, APOA2, APOA4, and APOC1 involved in the regulation of cholesterol esterification and phospholipid efflux (Figure 7). Higher levels of APOA1 have been correlated with protection from COVID-19 severity [27]. Furthermore, cholesterol esterification may counteract the normally exacerbating effect of cholesterol on coronavirus cytopathology [28]. Consequently, our results suggest that higher levels of some apolipoproteins in PCR– individuals may be associated with a vaccine-protective effect.

## 4. Conclusions

In summary, novel findings of the study include (a) characterization of Ig and non-Ig protein profiles in vaccinated uninfected (fully protected) and vaccinated SARS-CoV-2-infected (partially protected) individuals with identification of disease and protection-associated biomarkers; (b) identification of candidate-protective epitopes not only in SARS-CoV-2 RBD but also in glycoprotein M (ORF3a), membrane protein E, and nucleocapsid phosphoprotein N (ORF1ab); (c) analysis of autoantibody profiles that are associated with an unfavorable COVID-19 disease course even after vaccination; and (d) prediction on non-Ig serum biomarkers associated with vaccine-protective capacity or disease severity in vaccinated and SARS-CoV-2-infected individuals.

The main limitation of this study is that serum-proteomics analysis was conducted with five samples from each cohort, which may have reduced the effect of case-by-case differences in serum-protein representation. Nevertheless, the results of this study using a serum-proteomics approach to characterize host-associated factors to COVID-19-vaccine response suggest protective- and disease-associated mechanisms in vaccinated individuals. Despite differences in individual age, sex, vaccine provider, and doses, the results were consistent between different cohorts. These results may lead to studies with a higher number of individuals and including different vaccine formulations to improve vaccine efficacy and implementation against SARS-CoV-2.

## Figures and Tables

**Table 1:** Data on serum samples included in the analysis.

Lab, Proteome ID	Age (y/o)	Sex	Sample Date (*)	Vaccine Doses	SARS-CoV-2-Neutralizing Antibodies	Dates of Vaccination
**Cohort PCR**–						
16107663, C1	79	F	15.12.2021 (247 days)	Pfizer Pfizer Moderna	95.2%	13.04.2021 11.05.2021 11.11.2021
16107723, C2	54	M	15.12.2021 (323 days)	Pfizer Pfizer	32.0%	27.01.2021 22.02.2021
39385248, C3	60	F	15.12.2021 (323 days)	Pfizer Pfizer Moderna	95.3%	27.01.2021 22.02.2021 30.11.2021
39385665, C4	82	F	15.12.2021 (255 days)	Pfizer Pfizer Pfizer	92.4%	05.04.2021 26.04.2021 08.11.2021
39386122, C5	54	F	16.12.2021 (342 days)	Pfizer Pfizer	2.3% (negative)	09.01.2021 30.01.2021
**Cohort PCR+ Asymptomatic**						
16107241, A1	84	F	09.12.2021 (255 days)	Pfizer Pfizer	39.4%	30.03.2021 20.04.2021
1433003, A2	88	F	12.12.2021 (272 days)	Pfizer Pfizer Pfizer	95.6%	16.03.2021 06.04.2021 29.10.2021
161082999, A3	89	F	23.12.2021 (351 days)	Pfizer Pfizer Pfizer	51.4%	07.01.2021 28.01.2021 04.10.2021
1437141, A4	68	M	26.12.2021 (229 days)	Pfizer Pfizer Pfizer	94.1%	12.05.2021 04.06.2021 24.11.2021
88403647, A5	46	F	30.12.2021 (205 days)	Pfizer Pfizer	70.2%	09.06.2021 30.06.2021
**Cohort PCR+ Non-severe Hospital Discharge. Symptoms: fever, cough**						
1429191, L1	41	F	28.11.2021 (170 days)	Pfizer	95.6%	12.06.2021
1433753, L2	19	M	14.12.2021 (140 days)	Pfizer Pfizer	75.6%	28.07.2021 18.08.2021
1433789, L3	25	F	14.12.2021 (103 days)	Pfizer	45.0%	03.09.2021
1435300, L4	26	M	20.12.2021 (168 days)	Pfizer Pfizer	62.8%	06.07.2021 27.07.2021
1435504, L5	47	M	20.12.2021 (199 days)	Pfizer Pfizer	96.1%	05.06.2021 26.06.2021
**Cohort PCR+ Severe Hospitalized. Symptoms: pneumonia, diarrhea, body weakness**						
16105221, S1	79	M	10.11.2021 (209 days)	Pfizer Pfizer	7.3% (negative)	16.04.2021 07.05.2021
16106123, S2	77	F	22.11.2021 (223 days)	Pfizer Pfizer	94.6%	14.04.2021 05.05.2021
1431680, S3	91	M	08.12.2021 (251 days)	Pfizer Pfizer Pfizer	61.7%	03.03.2021 24.03.2021 03.11.2021
1432590, S4	73	M	10.12.2021 (229 days)	Pfizer Pfizer	95.5%	26.04.2021 17.05.2021
1434692, S5	75	F	17.12.2021 (225 days)	Pfizer Pfizer Moderna	92.9%	07.05.2021 28.05.2021 09.12.2021
**Cohort PCR+ ICU. Symptoms: severe bilateral pneumonia with acute respiratory distress**						
1432410, U1	52	F	10.12.2021 (194 days)	Janssen Moderna	95.0%	31.05.2021 23.11.2021
1434573, U2	52	M	17.12.2021 (201 days)	Janssen Moderna	94.7%	31.05.2021 23.11.2021

(*) Days between first vaccine dose and serum sampling are shown in parentheses.

## Data Availability

All data are available in the main text or the Supplementary Materials. The mass-spectrometry proteomics data have been deposited in the ProteomeXchange Consortium via the PRIDE partner repository with the dataset identifier PXD031969 and 10.6019/PXD031969.

## Supplementary Materials

The following supporting information can be downloaded at: https://www.mdpi.com/article/10.3390/molecules27185933/s1, Data File S1. Serum proteomics analysis; Data File S2. Analysis of immunoglobulin proteins underrepresented and overrepresented in infected cohorts when compared to PCR– individuals; Data File S3. Human-autoantibody general survey in PCR– and PCR+ cohorts; Data File S4. Analysis of selected non-immunoglobulin proteins underrepresented and overrepresented in infected cohorts when compared to PCR– individuals.

## Sample Availability

Samples of the compounds are available from the authors.

## References

[1] Adam D. (2022). The pandemic’s true death toll: Millions more than official counts. Nature.

[2] Chaudhary J.K., Yadav R., Chaudhary P.K., Maurya A., Kant N., Rugaie O.A., Haokip H.R., Yadav D., Roshan R., Prasad R. (2021). Insights into COVID-19 Vaccine development based on immunogenic structural proteins of SARS-CoV-2, host immune responses, and herd immunity. Cells.

[3] Ceacareanu A.C., Wintrob Z.A.P. (2021). Summary of COVID-19 vaccine-related reports in the vaccine adverse event reporting system. J. Res. Pharm. Pract..

[4] Adesokan A., Obeid M.A., Lawal A.F. (2022). SARS-CoV-2: Vaccinology and emerging therapeutics; challenges and future developments. Ther. Deliv..

[5] Mohapatra R.K., Kuppili S., Kumar Suvvari T., Kandi V., Behera A., Verma S., Kudrat-E-Zahan Biswal S.K., Al-Noor T.H., El-Ajaily M.M., Sarangi A.K. (2022). SARS-CoV-2 and its variants of concern including Omicron: Looks like a never ending pandemic. Chem. Biol. Drug Des..

[6] Li J., Wang Y., Liu Y., Zhang Z., Zhai Y., Dai Y., Wu Z., Nie X., Du L. (2022). Polymorphisms and mutations of ACE2 and TMPRSS2 genes are associated with COVID-19: A systematic review. Eur. J. Med. Res..

[7] Amiri-Dashatan N., Koushki M., Rezaei-Tavirani M. (2022). Mass spectrometry-based proteomics research to fight COVID-19: An expert review on hopes and challenges. OMICS.

[8] Villar M., Urra J.M., Rodríguez-Del-Río F.J., Artigas-Jerónimo S., Jiménez-Collados N., Ferreras-Colino E., Contreras M., Fernández de Mera I.G., Estrada-Peña A., Gortázar C. (2021). Characterization by quantitative serum proteomics of immune-related prognostic biomarkers for COVID-19 symptomatology. Front. Immunol..

[9] Kunik V., Ashkenazi S., Ofran Y. (2012). Paratome: An online tool for systematic identification of antigen-binding regions in antibodies based on sequence or structure. Nucleic Acids Res..

[10] D’Angelo S., Ferrara F., Naranjo L., Erasmus M.F., Hraber P., Bradbury A.R.M. (2018). Many routes to an Antibody Heavy-Chain CDR3: Necessary, yet insufficient, for specific binding. Front. Immunol..

[11] Taeschler P., Cervia C., Zurbuchen Y., Hasler S., Pou C., Tan Z., Adamo S., Raeber M.E., Bächli E., Rudiger A. (2022). Autoantibodies in COVID-19 correlate with antiviral humoral responses and distinct immune signatures. Allergy.

[12] Uchida M., Tashiro-Itoh T., Matsuda Y., Ishihara K., Asakura H. (1998). Autoantibodies against a 210 kDa glycoprotein of the nuclear pore complex as a prognostic marker in patients with primary biliary cirrhosis. J. Gastroenterol. Hepatol..

[13] Yeung M.L., Teng J.L.L., Jia L., Zhang C., Huang C., Cai J.P., Zhou R., Chan K.H., Zhao H., Zhu L. (2021). Soluble ACE2-mediated cell entry of SARS-CoV-2 via interaction with proteins related to the renin-angiotensin system. Cell.

[14] Miedema J., Schreurs M., van der Sar-van der Brugge S., Paats M., Baart S., Bakker M., Hoek R., Dik W.A., Endeman H., Van Der Velden V. (2021). Antibodies against Angiotensin II Receptor Type 1 and Endothelin A Receptor are associated with an unfavorable COVID19 disease course. Front. Immunol..

[15] Niu L., Wittrock K.N., Clabaugh G.C., Srivastava V., Cho M.W. (2021). A structural landscape of neutralizing antibodies against SARS-CoV-2 Receptor Binding Domain. Front. Immunol..

[16] Bastard P. (2022). Why do people die from COVID-19?. Science.

[17] Schiaffini R., Campana A., Deodati A., Peschiaroli E., Lanzillotta M.F., Fierabracci A. (2022). SARS-CoV-2 infection as possible downstream disease precipitator in autoantibody-positive insulin-dependent diabetes mellitus: A case report. Ital. J. Pediatr..

[18] Dotan A., David P., Arnheim D., Shoenfeld Y. (2022). The autonomic aspects of the post-COVID19 syndrome. Autoimmun. Rev..

[19] Juanes-Velasco P., Landeira-Viñuela A., García-Vaquero M.L., Lecrevisse Q., Herrero R., Ferruelo A., Góngora R., Corrales F., Rivas J.L., Lorente J.A. (2022). SARS-CoV-2 Infection Triggers Auto-Immune Response in ARDS. Front. Immunol..

[20] Afzali B., Noris M., Lambrecht B.N., Kemper C. (2022). The state of complement in COVID-19. Nat. Rev. Immunol..

[21] Ilias I., Diamantopoulos A., Botoula E., Athanasiou N., Zacharis A., Tsipilis S., Jahaj E., Vassiliou A.G., Vassiliadi D.A., Kotanidou A. (2021). COVID-19 and Growth Hormone/Insulin-Like Growth Factor 1: Study in critically and non-critically ill patients. Front. Endocrinol..

[22] Fan X., Yin C., Wang J., Yang M., Ma H., Jin G., Song M., Hu Z., Shen H., Hang D. (2021). Pre-diagnostic circulating concentrations of insulin-like growth factor-1 and risk of COVID-19 mortality: Results from UK Biobank. Eur. J. Epidemiol..

[23] Yoon I.S., Park H., Kwak H.W., Woo Jung Y., Nam J.H. (2017). Macrophage-derived insulin-like growth factor-1 affects influenza vaccine efficacy through the regulation of immune cell homeostasis. Vaccine.

[24] Weller R., Hueging K., Brown R.J.P., Todt D., Joecks S., Vondran F.W.R., Pietschmann T. (2017). Hepatitis C virus strain-dependent usage of Apolipoprotein E modulates assembly efficiency and specific infectivity of secreted virions. J. Virol..

[25] Hubacek J.A. (2021). Effects of selected inherited factors on susceptibility to SARS-CoV-2 infection and COVID-19 progression. Physiol. Res..

[26] Nuñez E., Orera I., Carmona-Rodríguez L., Paño J.R., Vázquez J., Corrales F.J. (2022). Mapping the Serum Proteome of COVID-19 Patients; Guidance for Severity Assessment. Biomedicines.

[27] Ulloque-Badaracco J.R., Hernandez-Bustamante E.A., Herrera-Añazco P., Benites-Zapata V.A. (2021). Prognostic value of apolipoproteins in COVID-19 patients: A systematic review and meta-analysis. Travel Med. Infect. Dis..

[28] Cervin M., Anderson R. (1991). Modulation of coronavirus-mediated cell fusion by homeostatic control of cholesterol and fatty acid metabolism. J. Med. Virol..

