# Supplementary material for: Correlates with Vaccine Protective Capacity and COVID-19 Disease Symptoms Identified by Serum Proteomics in Vaccinated Individuals
Source: Molecules. 2022 Sep 13;27(18):5933. doi: 10.3390/molecules27185933 (PMC9500703; doi:10.3390/molecules27185933)
Supplement: Supplementary file 1 [file molecules-27-05933-s001.zip › Data file S2.pdf]

Data file S2. Analysis of immunoglobulin proteins underrepresented and overrepresented in infected cohorts when compared to PCR- individuals.

Part 1. Analysis of immunoglobulin proteins underrepresented in infected cohorts when compared to PCR- individuals.

Analytical workflow

1. Select immunoglobulin protein sequences underrepresented in infected cohorts.
2. Protein BLAST sequence alignment against non-redundant protein database(nr)using compositional matrix adjustment (https://blast.ncbi.nlm.nih.gov/Blast.cgi?PROGRAM=blastp&PAGE_TYPE=BlastSearch&LINK_LOC=blasthome).
3. Identification of anti-SARS-CoV-2 immunoglobulin sequences aligned. Criteria: Score > 160 bits, Identity > 60%.
4. Paratome (http://www.ofranlab.org) identification of antigen binding regions.
5. Protein BLAST sequence alignment against SARS-CoV-2 using compositional matrix adjustment (https://blast.ncbi.nlm.nih.gov/Blast.cgi?PROGRAM=blastp&PAGE_TYPE=BlastSearch&LINK_LOC=blasthome).
6. Identification of SARS-CoV-2 sequences aligned. Criteria: Score > 15 bits, Identity > 60%.
7. Identification of correlates of vaccine-induced antibody protective epitopes with emphasis on SARS-CoV-2 Spike S RBD domain.

Input data:

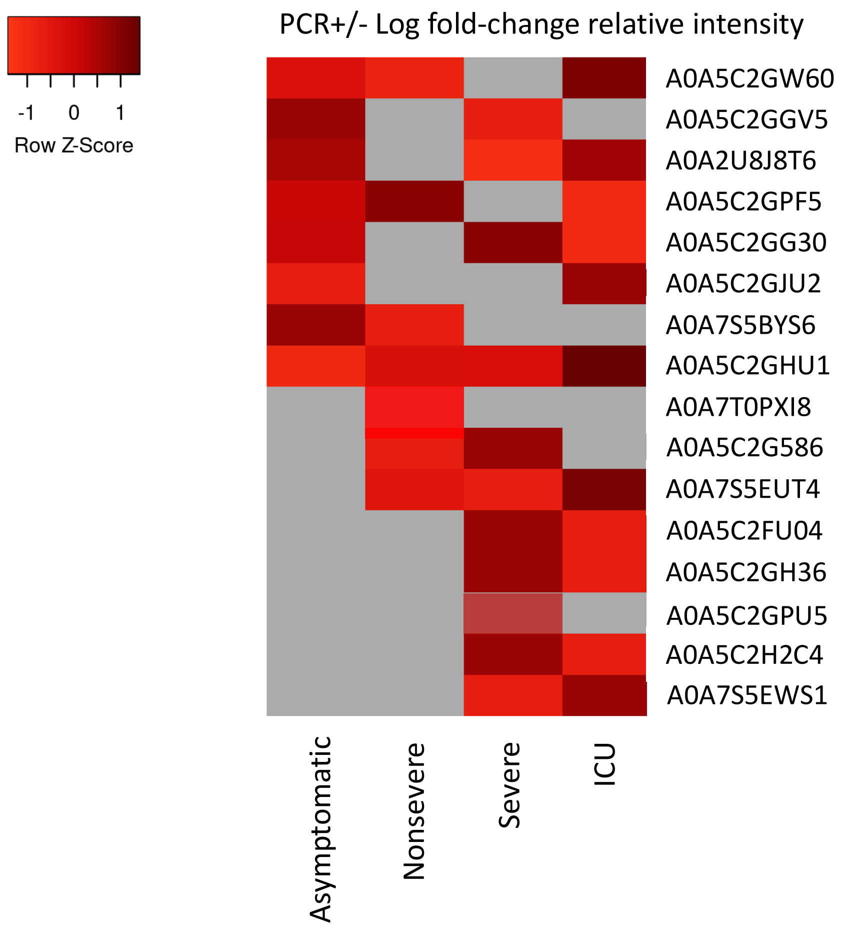

A0A5C2GW60

>tr|A0A5C2GW60|A0A5C2GW60_HUMAN IG c1795_light_IGKV3-15_IGKJ4 (Fragment) OS=Homo sapiens OX=9606 PE=2 SV=1
QIVMTQSPASLSVFPGDSATLFCRASQRIGGNLAWYQQRPGQAPRLLIYGSSNRAAGVPP
RFSGSGSGTDFTLTINSLRSEDYAVYYCQQHHAWPPAFGGGTKVEIK

BLAST Sequence aligment against nr

Query: tr|A0A5C2GW60|A0A5C2GW60_HUMAN IG c1795_light_IGKV3-15_IGKJ4 (Fragment) OS=Homo sapiens OX=9606 PE=2 SV=1 Query ID: lcl|Query_811367
Length: 107

>anti-SARS-CoV-2 immunoglobulin kappa light chain variable region, partial [Homo sapiens]
Sequence ID: QJU69756.1 Length: 109
>immunoglobulin light chain variable region, partial [Homo sapiens]
Sequence ID: QYF06493.1 Length: 109
Range 1: 1 to 109

Score:176 bits(445), Expect:1e-54,
Method:Compositional matrix adjust.,
Identities:83/109(76%), Positives:97/109(88%), Gaps:2/109(1%)

```
Query  1    QIVMTQSPASLSVFPGDSATLFCRASQRIGGNLAWYQQRPGQAPRLLIYGSSNRAAGVPP  60
            +IVMTQSPA+LSV PG+ ATL CRASQ +  NLAWYQQ+PGQAPRLLIYG+S RA G+P
Sbjct  1    EIVMTQSPATLSVSPGERATLSCRASQSVSSNLAWYQQKPGQAPRLLIYGASTRATGIPA  60

Query  61   RFSGSGSGTDFTLTINSLRSEDYAVYYCQQHHAWPPA--FGGGTKVEIK  107
            RFSGSGSGT+FTLTI+SL+SED+AVYYCQQ++ WPP+  FGGGTKVEIK
Sbjct  61   RFSGSGSGTEFTLTISSLQSEDFAVYYCQQYNNWPPSLTFGGGTKVEIK  109
```

Paratome analysis

paratome_1_seq_27691_107_bp
ABR L1: QRIGGNLA (27-34)
ABR L2: LLIYGSSNRAA (46-56)
ABR L3: QQHHAWPPA (89-97)

BLAST Sequence aligment against SARS-CoV-2

ABR L1: QRIGGNLA (27-34)
No significant similarity found.

ABR L2: LLIYGSSNRAA (46-56)
>surface glycoprotein [Severe acute respiratory syndrome coronavirus 2]
Sequence ID: UJH96504.1 Length: 1271
Range 1: 751 to 764

Score:21.4 bits(43), Expect:6037,
Method:,
Identities:9/14(64%), Positives:9/14(64%), Gaps:4/14(28%)

```
Query  1    LLIYGSS----NRA  10
            LL YGSS    NRA
Sbjct  751  LLQYGSSCTQLNRA  764
```

ABR L3: QQHHAWPPA (89-97)
No significant similarity found.

A0A5C2GGV5

>tr|A0A5C2GGV5|A0A5C2GGV5_HUMAN IG c110_light_IGKV4-1_IGKJ4 (Fragment) OS=Homo sapiens OX=9606 PE=2 SV=1
DIVMTQSPDSLAVSLGERATINCKSSQNILYRANNKNYLAWYQQKPGQPPKLLVSWASTR
ESGVPDRFNGSGSGTDFNLTISSLQAEDVAVYSCQQYYSTPLTFGGGTKVEIK

BLAST Sequence aligment against nr

Query: tr|A0A5C2GGV5|A0A5C2GGV5_HUMAN IG c110_light_IGKV4-1_IGKJ4 (Fragment) OS=Homo sapiens OX=9606 PE=2 SV=1 Query ID: lcl|Query_252275
Length: 113

>anti-SARS-CoV-2 immunoglobulin light chain variable region, partial [Homo sapiens]
Sequence ID: QTX15727.1 Length: 113
Range 1: 1 to 113

Score:218 bits(554), Expect:5e-71,
Method:Compositional matrix adjust.,
Identities:102/113(90%), Positives:109/113(96%), Gaps:0/113(0%)

```
Query  1    DIVMTQSPDSLAVSLGERATINCKSSQNILYRANNKNYLAWYQQKPGQPPKLLVSWASTR  60
            DIVMTQSPDSLAVSLGERATINCKSSQ++L+ +NNKNY+AWYQQKPGQPPKLL+ WASTR
Sbjct  1    DIVMTQSPDSLAVSLGERATINCKSSQSVLHSSNNKNYVAWYQQKPGQPPKLLIYWASTR  60

Query  61   ESGVPDRFNGSGSGTDFNLTISSLQAEDVAVYSCQQYYSTPLTFGGGTKVEIK  113
            ESGVPDRF+GSGSGTDF LTISSLQAEDVAVY CQQYYSTPLTFGGGTKVEIK
Sbjct  61   ESGVPDRFSGSGSGTDFTLTISSLQAEDVAVYHCQQYYSTPLTFGGGTKVEIK  113
```

Paratome analysis

paratome_1_seq_27871_113_bp
ABR L1: QNILYRANNKNYLA (27-40)
ABR L2: LLVSWASTRES (52-62)
ABR L3: QQYYSTPL (95-102)

BLAST Sequence aligment against SARS-CoV-2

ABR L1: QNILYRANNKNYLA (27-40)
>ORF10 protein [Severe acute respiratory syndrome coronavirus 2]
Sequence ID: UDE38504.1 Length: 38
>ORF10 protein [Severe acute respiratory syndrome coronavirus 2]
Sequence ID: UDN73246.1 Length: 38
>ORF10 protein [Severe acute respiratory syndrome coronavirus 2]
Sequence ID: UEF92301.1 Length: 38
>ORF10 protein [Severe acute respiratory syndrome coronavirus 2]
Sequence ID: UEI16040.1 Length: 38
>ORF10 protein [Severe acute respiratory syndrome coronavirus 2]
Sequence ID: UEI16377.1 Length: 38
Range 1: 18 to 28

Score:24.8 bits(51), Expect:619,
Method:,
Identities:7/11(64%), Positives:7/11(63%), Gaps:0/11(0%)

```
Query  4   LYRANNKNYLA  14
           LYR N  NY A
Sbjct  18  LYRMNSRNYIA  28
```

>surface glycoprotein [Severe acute respiratory syndrome coronavirus 2]
Sequence ID: UJT26857.1 Length: 1270

Range 1: 910 to 918

Score:21.4 bits(43), Expect:10532,
Method:,
Identities:7/10(70%), Positives:8/10(80%), Gaps:1/10(10%)

```
Query  1    QNILYRANNK  10
            QN+LY AN K
Sbjct  910  QNVLY-ANQK  918
```

ABR L2: LLVSWASTRES (52-62)

>ORF1ab polyprotein, partial [Severe acute respiratory syndrome coronavirus 2]
Sequence ID: QZK57390.1 Length: 7077
Range 1: 4948 to 4956

Score:19.7 bits(39), Expect:25032,
Method:,
Identities:6/9(67%), Positives:6/9(66%), Gaps:0/9(0%)

```
Query  1     LLVSWASTR  9
             LL S A TR
Sbjct  4948  LLXSXAATR  4956
```

ABR L3: QQYYSTPL (95-102)

>ORF3a protein [Severe acute respiratory syndrome coronavirus 2]
Sequence ID: UIJ25215.1 Length: 275
Range 1: 213 to 219

Score:19.7 bits(39), Expect:13007,
Method:,
Identities:6/7(86%), Positives:6/7(85%), Gaps:0/7(0%)

```
Query  2    QYYSTPL  8
            Q YSTPL
Sbjct  213  QLYSTPL  219
```

>ORF1ab polyprotein [Severe acute respiratory syndrome coronavirus 2]
Sequence ID: QYZ75299.1 Length: 7096
Range 1: 4171 to 4175

Score:18.0 bits(35), Expect:53647,
Method:,
Identities:4/5(80%), Positives:4/5(80%), Gaps:0/5(0%)

```
Query  3     YYSTP  7
             YY TP
Sbjct  4171  YYNTP  4175
```

A0A2U8J8T6

>tr|A0A2U8J8T6|A0A2U8J8T6_HUMAN Ig heavy chain variable region (Fragment) OS=Homo sapiens OX=9606 GN=IgH PE=2 SV=1
GSGYSFSGYWIVWVRQMPGKGLEWMGLIYPDDSNTRYSPSFQGQVTFSADKSITTAYLQW
SSLRASDTAIYYCARLSGPNRIMRAHWFDPWGQGTLVTVSS

BLAST Sequence aligment against nr

Query: tr|A0A2U8J8T6|A0A2U8J8T6_HUMAN Ig heavy chain variable region (Fragment) OS=Homo sapiens OX=9606 GN=IgH PE=2 SV=1 Query ID: lcl|Query_416432 Length: 101

>anti-SARS-CoV-2 immunoglobulin heavy chain variable region, partial [Homo sapiens]
Sequence ID: UKB89132.1 Length: 126
Range 1: 24 to 126

Score:161 bits(408), Expect:7e-49,
Method:Compositional matrix adjust.,
Identities:82/103(80%), Positives:90/103(87%), Gaps:2/103(1%)

```
Query  1    GSGYSFSGYWIVWVRQMPGKGLEWMGLIYPDDSNTRYSPSFQGQVTFSADKSITTAYLQW  60
            GSGYSF+ YWI WVRQMPGKGLEWMG+IYP DS+TRYSPSFQGQVT SADKSI+TAYLQW
Sbjct  24   GSGYSFTSYWIGWVRQMPGKGLEWMGIIYPGDSDTRYSPSFQGQVTISADKSISTAYLQW  83

Query  61   SSLRASDTAIYYCAR-LSGPNRIM-RAHWFDPWGQGTLVTVSS  101
            SSL+ASDTA+YYCAR +SG +       WFDPWGQGTLVTVSS
Sbjct  84   SSLKASDTAMYYCARHMSGTHSSGWYERWFDPWGQGTLVTVSS  126
```

Paratome analysis

paratome_1_seq_28012_101_bp
Could not find ABR1
ABR H2: WMGLIYPDDSNTRY (24-37)
ABR H3: RLSGPNRIMRAHWFDP (75-90)

BLAST Sequence aligment against SARS-CoV-2

ABR H2: WMGLIYPDDSNTRY (24-37)
>Chain B, S2X303 Fab heavy chain [Severe acute respiratory syndrome coronavirus 2]
Sequence ID: 7SOE_B Length: 125
>Chain E, S2X303 Fab heavy chain [Severe acute respiratory syndrome coronavirus 2]
Sequence ID: 7SOE_E Length: 125
>Chain H, S2X303 Fab heavy chain [Severe acute respiratory syndrome coronavirus 2]
Sequence ID: 7SOE_H Length: 125
Range 1: 49 to 54

Score:18.9 bits(37), Expect:86006,
Method:,
Identities:4/6(67%), Positives:5/6(83%), Gaps:0/6(0%)

```
Query  1   WMGLIY  6
           W+ LIY
Sbjct  49  WLALIY  54
```

ABR H3: RLSGPNRIMRAHWFDP (75-90)

>ORF1a polyprotein [Severe acute respiratory syndrome coronavirus 2]
Sequence ID: UCK56015.1 Length: 4405

```
Range 1: 3662 to 3668

Score:21.0 bits(42), Expect:20697,
Method:,
Identities:6/9(67%), Positives:6/9(66%), Gaps:2/9(22%)

Query  7     RIMRAHWFD  15
             RIMR  W D
Sbjct  3662  RIMR--WLD  3668

>surface glycoprotein, partial [Severe acute respiratory syndrome
coronavirus 2]
Sequence ID: QTM47912.1 Length: 1259
Range 1: 1086 to 1089

Score:18.9 bits(37), Expect:118712,
Method:,
Identities:4/4(100%), Positives:4/4(100%), Gaps:0/4(0%)

Query  11    AHWF  14
             AHWF
Sbjct  1086  AHWF  1089
```

A0A5C2GPF5

>tr|A0A5C2GPF5|A0A5C2GPF5_HUMAN IG c1256_heavy_IGHV3-23_IGHD3-3_IGHJ6 (Fragment) OS=Homo sapiens OX=9606 PE=2 SV=1
EVQLLESGGGRLHPGGSLRLSCAASGFTFNNYAMSWVRQAPEKGLEWVSAVSGSGASTYY
ADSVKGRFTISRDNSKNTLYLQMSSLRVEDTAVYYCAKVARLTVFGVVNTGHFMDVWGKG
TTVTVSS

BLAST Sequence aligment against nr

Query: tr|A0A5C2GPF5|A0A5C2GPF5_HUMAN IG c1256_heavy_IGHV3-23_IGHD3-3_IGHJ6 (Fragment) OS=Homo sapiens OX=9606 PE=2 SV=1 Query ID: lcl|Query_615149
Length: 127

>anti-SARS-CoV-2 spike protein immunoglobulin heavy chain variable region, partial [Homo sapiens]
Sequence ID: QKY76482.1 Length: 124
Range 1: 1 to 124

Score:204 bits(519), Expect:2e-65,
Method:Compositional matrix adjust.,
Identities:101/127(80%), Positives:109/127(85%), Gaps:3/127(2%)

```
Query  1    EVQLLESGGGRLHPGGSLRLSCAASGFTFNNYAMSWVRQAPEKGLEWVSAVSGSGASTYY  60
            EVQLL+SGGG + PGGSLRLSCAASGFTF NYAMSWVRQAP KGLEWVSA+SGSG +TYY
Sbjct  1    EVQLLQSGGGLVQPGGSLRLSCAASGFTFRNYAMSWVRQAPGKGLEWVSAISGSGGTTYY  60

Query  61   ADSVKGRFTISRDNSKNTLYLQMSSLRVEDTAVYYCAKVARLTVFGVVNTGHFMDVWGKG  120
            ADSVKGRFTISRDNSKNTLYLQM+SLR EDTAVYYCAK  R+T+  VV      D WG+G
Sbjct  61   ADSVKGRFTISRDNSKNTLYLQMNSLRAEDTAVYYCAKNERITMLVVVT---LFDYWGQG  117

Query  121  TTVTVSS  127
            T VTVSS
Sbjct  118  TLVTVSS  124
```

Paratome analysis

paratome_1_seq_28214_127_bp
ABR H1: FTFNNYAMS (27-35)
ABR H2: WVSAVSGSGASTYY (47-60)
ABR H3: KVARLTVFGVVNTGHFMDV (98-116)

BLAST Sequence aligment against SARS-CoV-2

ABR H1: FTFNNYAMS (27-35)
>ORF1ab polyprotein [Severe acute respiratory syndrome coronavirus 2]
Sequence ID: UKG54270.1 Length: 7089
Range 1: 4461 to 4467

Score:20.6 bits(41), Expect:8026,
Method:,
Identities:5/7(71%), Positives:5/7(71%), Gaps:0/7(0%)

```
Query  2     TFNNYAM  8
             TF NY M
Sbjct  4461  TFSNYQM  4467
```

ABR H2: WVSAVSGSGASTYY (47-60)

>surface glycoprotein, partial [Severe acute respiratory syndrome coronavirus 2]
Sequence ID: QRX36841.1 Length: 682
Range 1: 256 to 266

```
Score:21.8 bits(44), Expect:7419,
Method:,
Identities:8/11(73%), Positives:8/11(72%), Gaps:2/11(18%)

Query  6    SG--SGASTYY  14
            SG  SGAS YY
Sbjct  256  SGWTSGASAYY  266
```

ABR H3: KVARLTVFGVVNTGHFMDV (98-116)

```
>ORF1ab polyprotein, partial [Severe acute respiratory syndrome coronavirus
2]
Sequence ID: UIQ72354.1 Length: 6975
Range 1: 6338 to 6346

Score:28.2 bits(59), Expect:88,
Method:,
Identities:8/9(89%), Positives:8/9(88%), Gaps:0/9(0%)

Query  8     FGVVNTGHF  16
             F VVNTGHF
Sbjct  6338  FNVVNTGHF  6346
```

A0A5C2GG30

>tr|A0A5C2GG30|A0A5C2GG30_HUMAN IG c723_heavy_IGHV4-39_IGHD6-6_IGHJ3 (Fragment) OS=Homo sapiens OX=9606 PE=2 SV=1
QVQLQESGPGLVKPSETLSLTCTVSGYYISSGYYWGCIRQPPGKGLEWIGSIHHSGSTYY
NPSLKSRVTISVDTSKNQFSLKLRSVTATDTAVYYCARRLPEQLVGSDAFDIWGQGTMVT
VSS

BLAST Sequence aligment against nr

Query: tr|A0A5C2GG30|A0A5C2GG30_HUMAN IG c723_heavy_IGHV4-39_IGHD6-6_IGHJ3 (Fragment) OS=Homo sapiens OX=9606 PE=2 SV=1 Query ID: lcl|Query_141113
Length: 123

>anti-SARS-CoV-2 immunoglobulin heavy chain variable region, partial [Homo sapiens]
Sequence ID: UKB89162.1 Length: 123
Range 1: 1 to 123

Score:186 bits(471), Expect:4e-58,
Method:Compositional matrix adjust.,
Identities:105/124(85%), Positives:111/124(89%), Gaps:2/124(1%)

```
Query  1    QVQLQESGPGLVKPSETLSLTCTVsgyyissgyyw-gCIRQPPGKGLEWIGSIHHSGSTY  59
            Q+QLQESGPGLVKPSETLSLTCTVSG  ISS  Y+ G IRQPPGKGLEWIGSI++SGSTY
Sbjct  1    QLQLQESGPGLVKPSETLSLTCTVSGGSISSSSYYWGWIRQPPGKGLEWIGSIYYSGSTY  60

Query  60   YNPSLKSRVTISVDTSKNQFSLKLRSVTATDTAVYYCARRLPEQLVGSDAFDIWGQGTMV  119
            YNPSLKSRVTISVDTSKNQFSLKL SVTA DTAVYYCAR+   +L G DAFDIWGQGTMV
Sbjct  61   YNPSLKSRVTISVDTSKNQFSLKLSSVTAADTAVYYCARQSSPKL-GDDAFDIWGQGTMV  119

Query  120  TVSS  123
            TVSS
Sbjct  120  TVSS  123
```

Paratome analysis

paratome_1_seq_28371_123_bp
ABR H1: YYISSGYYWGC (27-37)
ABR H2: WIGSIHHSGSTYY (48-60)
ABR H3: RRLPEQLVGSDAFDI (98-112)

BLAST Sequence aligment against SARS-CoV-2

ABR H1: YYISSGYYWGC (27-37)
>ORF3a protein [Severe acute respiratory syndrome coronavirus 2]
Sequence ID: QTW51530.1 Length: 275
Range 1: 206 to 212

Score:20.6 bits(41), Expect:12304,
Method:,
Identities:5/7(71%), Positives:5/7(71%), Gaps:0/7(0%)

```
Query  2    YISSGYY  8
            YI S YY
Sbjct  206  YITSDYY  212
```

ABR H2: WIGSIHHSGSTYY (48-60)
>surface glycoprotein [Severe acute respiratory syndrome coronavirus 2]
Sequence ID: UIL01164.1 Length: 1271
Range 1: 64 to 72

Score:19.7 bits(39), Expect:36472,
Method:,

```
Identities:5/9(56%), Positives:5/9(55%), Gaps:0/9(0%)

Query  1   WIGSIHHSG  9
           W   IH SG
Sbjct  64  WXXAIHXSG  72

ABR H3: RRLPEQLVGSDAFDI (98-112)

>nucleocapsid phosphoprotein [Severe acute respiratory syndrome coronavirus
2]
Sequence ID: UHO63712.1 Length: 419
Range 1: 276 to 284

Score:21.4 bits(43), Expect:12409,
Method:,
Identities:6/9(67%), Positives:6/9(66%), Gaps:0/9(0%)

Query  1    RRLPEQLVG  9
            RR PEQ  G
Sbjct  276  RRXPEQXXG  284
```

A0A5C2GJU2

>tr|A0A5C2GJU2|A0A5C2GJU2_HUMAN IG c195_heavy_IGHV3-30_IGHD1-26_IGHJ4 (Fragment) OS=Homo sapiens OX=9606 PE=2 SV=1
QVQLVESGGGVVQPGRSLRLSCAASGFSFSNYGMHWVRQAPGKGLEWLSVISYDGNIKYY
VDSVKGRFTISRDNSKNTLYLEMNSLRAEDSAVYYCAKGVEWELLKYLDSWGHGTLVTVSS

BLAST Sequence aligment against nr

Query: tr|A0A5C2GJU2|A0A5C2GJU2_HUMAN IG c195_heavy_IGHV3-30_IGHD1-26_IGHJ4 (Fragment) OS=Homo sapiens OX=9606 PE=2 SV=1 Query ID: lcl|Query_80258
Length: 121

>anti-SARS-CoV-2 spike protein immunoglobulin heavy chain variable region, partial [Homo sapiens]
Sequence ID: QKY76580.1 Length: 120
Range 1: 1 to 120

Score:212 bits(540), Expect:8e-69,
Method:Compositional matrix adjust.,
Identities:103/121(85%), Positives:110/121(90%), Gaps:1/121(0%)

```
Query  1    QVQLVESGGGVVQPGRSLRLSCAASGFSFSNYGMHWVRQAPGKGLEWLSVISYDGNIKYY  60
            QVQLVESGGGVVQPGRSLRLSCAASGF+FSNYGMHWVRQAPGKGLEW++VISYDG  KYY
Sbjct  1    QVQLVESGGGVVQPGRSLRLSCAASGFTFSNYGMHWVRQAPGKGLEWVAVISYDGTNKYY  60

Query  61   VDSVKGRFTISRDNSKNTLYLEMNSLRAEDSAVYYCAKGVEWELLKYLDSWGHGTLVTVS  120
             DSVKGRFTISRDNSKNTLYL+MNSLRA+D+AVYYCAKG     L + DSWG GTLVTVS
Sbjct  61   ADSVKGRFTISRDNSKNTLYLQMNSLRADDTAVYYCAKG-RGNYLTFFDSWGQGTLVTVS  119

Query  121  S  121
            S
Sbjct  120  S  120
```

Paratome analysis

paratome_1_seq_6084_121_bp
ABR H1: FSFSNYGMH (27-35)
ABR H2: WLSVISYDGNIKYYV (47-61)
ABR H3: KGVEWELLKYLDS (98-110)

BLAST Sequence aligment against SARS-CoV-2

ABR H1: FSFSNYGMH (27-35)

>ORF1ab polyprotein [Severe acute respiratory syndrome coronavirus 2]
Sequence ID: UFG71429.1 Length: 7096
Range 1: 3156 to 3160

Score:19.7 bits(39), Expect:16289,
Method:,
Identities:5/5(100%), Positives:5/5(100%), Gaps:0/5(0%)

```
Query  2     SFSNY  6
             SFSNY
Sbjct  3156  SFSNY  3160
```

Range 2: 4469 to 4472

Score:17.2 bits(33), Expect:139431,
Method:,
Identities:4/4(100%), Positives:4/4(100%), Gaps:0/4(0%)

```
Query  3     FSNY  6
             FSNY
Sbjct  4469  FSNY  4472
```

ABR H2: WLSVISYDGNIKYYV (47-61)
>ORF1a polyprotein [Severe acute respiratory syndrome coronavirus 2]
Sequence ID: UHN63799.1 Length: 4405
Range 1: 1550 to 1557

Score:18.9 bits(37), Expect:102501,
Method:,
Identities:6/9(67%), Positives:6/9(66%), Gaps:1/9(11%)

```
Query  4     VISYDGNIK  12
             VI YD N K
Sbjct  1550  VITYD-NLK  1557
```

ABR H3: KGVEWELLKYLDS (98-110)

>ORF1ab polyprotein [Severe acute respiratory syndrome coronavirus 2]
Sequence ID: UAT13595.1 Length: 7096
Range 1: 4658 to 4665

Score:26.9 bits(56), Expect:92,
Method:,
Identities:6/8(75%), Positives:8/8(100%), Gaps:0/8(0%)

```
Query  3     VEWELLKY  10
             +EW+LLKY
Sbjct  4658  IEWDLLKY  4665
```

A0A7S5BYS6

```
>tr|A0A7S5BYS6|A0A7S5BYS6_HUMAN IGH c384_heavy_IGHV1-69_IGHD4-23_IGHJ6
(Fragment) OS=Homo sapiens OX=9606 PE=2 SV=1
QVQLVQSAAEVKKPGSSVKVSCKASGGTFSGYTVSWVRQAPGQGLEWMGAVIPVYGTAQY
AWKFQGRVTISADESTSTAYLDLSRLTSEDTAVYYCARGDNGGLGYWYFYMDVWGRGTTVIVSS
```

BLAST Sequence aligment against nr

```
>anti-SARS-CoV-2 spike protein immunoglobulin heavy chain variable region,
partial [Homo sapiens]
Sequence ID: QKY76551.1 Length: 124
Range 1: 1 to 124

Score:193 bits(490), Expect:6e-61,
Method:Compositional matrix adjust.,
Identities:95/124(77%), Positives:105/124(84%), Gaps:0/124(0%)

Query  1    QVQLVQSAAEVKKPGSSVKVSCKASGGTFSGYTVSWVRQAPGQGLEWMGAVIPVYGTAQY  60
            QVQLVQS AEVKKPGSSVKVSCKASGGTF  Y +SWVRQAPGQGLEWMG  IP+ GT  Y
Sbjct  1    QVQLVQSGAEVKKPGSSVKVSCKASGGTFHNYAISWVRQAPGQGLEWMGGFIPILGTTNY  60

Query  61   AWKFQGRVTISADESTSTAYLDLSRLTSEDTAVYYCARGDNGGLGYWYFYMDVWGRGTTV  120
            A KFQGRVTI+ADESTSTAY++LS L SEDTAVYYCAR +  G+  +Y+ MDVWG+GTTV
Sbjct  61   AQKFQGRVTITADESTSTAYMELSSLRSEDTAVYYCARVEGEGVDSYYYGMDVWGQGTTV  120

Query  121  IVSS  124
             VSS
Sbjct  121  TVSS  124
```

Paratome analysis

```
paratome_1_seq_6446_124_bp
ABR H1: GTFSGYTVS (27-35)
ABR H2: WMGAVIPVYGTAQY (47-60)
ABR H3: RGDNGGLGYWYFYMDV (98-113)
```

BLAST Sequence aligment against SARS-CoV-2

```
ABR H1: GTFSGYTVS (27-35)
>ORF1ab polyprotein [Severe acute respiratory syndrome coronavirus 2]
Sequence ID: UFU96319.1 Length: 7096
Range 1: 1334 to 1339

Score:19.7 bits(39), Expect:16424,
Method:,
Identities:5/6(83%), Positives:5/6(83%), Gaps:0/6(0%)

Query  3     FSGYTV  8
             F GYTV
Sbjct  1334  FNGYTV  1339

ABR H2: WMGAVIPVYGTAQY (47-60)
>surface glycoprotein [Severe acute respiratory syndrome coronavirus 2]
Sequence ID: UAJ56903.1 Length: 1270
Range 1: 149 to 160

Score:21.0 bits(42), Expect:15102,
Method:,
Identities:6/12(50%), Positives:6/12(50%), Gaps:0/12(0%)

Query  1    WMGAVIPVYGTA  12
            WM  V  VY  A
Sbjct  149  WMESVFRVYSSA  160
```

ABR H3: RGDNGGLGYWYFYMDV (98-113)
>nucleocapsid phosphoprotein, partial [Severe acute respiratory syndrome coronavirus 2]
Sequence ID: UBE05898.1 Length: 416
Range 1: 95 to 111

Score:22.7 bits(46), Expect:5107,
Method:,
Identities:7/17(41%), Positives:9/17(52%), Gaps:4/17(23%)

```
Query  1    RGDNGGLGY----WYFY  13
            RG +G +      WYFY
Sbjct  95   RGGDGXMXXXXXRWYFY  111
```

A0A5C2GHU1

QLRLVQSGGEVRKPGASVKVSCQTSGYSFTNFAVSWVRQAPGHGLEWMGRISGSNGVADYAQDFQDRVTMTTDTV
TSTAYMEMRSLTSDDTAIYYCARDRRLDYYENSAYFLGHDGFDIWGQGTMVTVLS

Ig Heavy variable >tr|A0A5C2GHU1|A0A5C2GHU1_HUMAN IG c789_heavy_IGHV1-18_IGHD3-22_IGHJ3 (Fragment) OS=Homo sapiens OX=9606 PE=2 SV=1

BLAST Sequence aligment against nr

Query: tr|A0A5C2GHU1|A0A5C2GHU1_HUMAN IG c789_heavy_IGHV1-18_IGHD3-22_IGHJ3 (Fragment) OS=Homo sapiens OX=9606 PE=2 SV=1 Query ID: lcl|Query_540851
Length: 130

>anti-SARS-CoV-2 immunoglobulin heavy chain variable region, partial [Homo sapiens]
Sequence ID: UKB89114.1 Length: 131
Range 1: 1 to 131
Score:161 bits(408), Expect:3e-48,
Method:Compositional matrix adjust.,
Identities:89/131(68%), Positives:105/131(80%), Gaps:1/131(0%)

```
Query  1    QLRLVQSGGEVRKPGASVKVSCQTSGYSFTNFAVSWVRQAPGHGLEWMGRISGSNGVADY  60
            Q++LVQSG EV+KPGASVKVSC+ SGY+FT++ +SWVRQAPG GLEWMG IS  NG  +Y
Sbjct  1    QVQLVQSGAEVKKPGASVKVSCKASGYTFTSYGISWVRQAPGQGLEWMGWISAYNGNTNY  60

Query  61   AQDFQDRVtmttdtvtstAYMEMRSLTSDDTAIYYCARDRRLDYYENSAYF-LGHDGFDI  119
            AQ  Q RVTMTTDT TSTAYME+RSL SDDTA+YYCARD    YY++S Y+    + FD
Sbjct  61   AQKLQGRVTMTTDTSTSTAYMELRSLRSDDTAVYYCARDGTSHYYDSSGYYGADRNWFDP  120

Query  120  WGQGTMVTVLS  130
            WGQGT+VTV S
Sbjct  121  WGQGTLVTVSS  131
```

>anti-SARS-CoV-2 immunoglobulin heavy chain variable region, partial [Homo sapiens]
Sequence ID: QTX15776.1 Length: 122
Range 1: 1 to 122

Score:161 bits(407), Expect:3e-48,
Method:Compositional matrix adjust.,
Identities:89/130(68%), Positives:102/130(78%), Gaps:8/130(6%)

```
Query  1    QLRLVQSGGEVRKPGASVKVSCQTSGYSFTNFAVSWVRQAPGHGLEWMGRISGSNGVADY  60
            +++LVQSG EV+KPGASVKVSC+ SGY+FT++ +SWVRQAPG GLEWMG IS  NG  +Y
Sbjct  1    EVQLVQSGAEVKKPGASVKVSCKASGYTFTSYGISWVRQAPGQGLEWMGWISAYNGNTNY  60

Query  61   AQDFQDRVtmttdtvtstAYMEMRSLTSDDTAIYYCARDRRLDYYENSAYFLGHDGFDIW  120
            AQ  Q RVTMTTDT TSTAYME+RSL SDDTA+YYCARD R           GHD FDIW
Sbjct  61   AQKLQGRVTMTTDTSTSTAYMELRSLRSDDTAVYYCARDTRGR--------GGHDAFDIW  112

Query  121  GQGTMVTVLS  130
            GQGTMVT+ S
Sbjct  113  GQGTMVTISS  122
```

>anti-SARS-CoV-2 spike protein immunoglobulin heavy chain variable region, partial [Homo sapiens]
Sequence ID: QKY76564.1 Length: 128
Range 1: 1 to 128

Score:160 bits(404), Expect:9e-48,
Method:Compositional matrix adjust.,
Identities:89/130(68%), Positives:102/130(78%), Gaps:2/130(1%)

```
Query  1    QLRLVQSGGEVRKPGASVKVSCQTSGYSFTNFAVSWVRQAPGHGLEWMGRISGSNGVADY  60
            Q++LVQSG EV+KPGASVKVSC+ SGY+FT++ +SWVRQAPG GLEWMG IS  NG  +Y
Sbjct  1    QVQLVQSGAEVKKPGASVKVSCKASGYTFTSYVISWVRQAPGQGLEWMGWISAYNGNTNY  60

Query  61   AQDFQDRVtmttdtvtstAYMEMRSLTSDDTAIYYCARDRRLDYYENSAYFLGHDGFDIW  120
            AQ  Q RVTMTTDT TSTAYME+RSL SDDTA+YYCARD+   YY  S     H G D+W
Sbjct  61   AQKLQGRVTMTTDTSTSTAYMELRSLRSDDTAVYYCARDQGPTYYYGSG--SPHYGMDVW  118

Query  121  GQGTMVTVLS  130
            GQGT VTV S
Sbjct  119  GQGTTVTVSS  128
```

Paratome analysis

paratome_1_seq_12087_130_bp
ABR H1: YSFTNFAVS (27-35)
ABR H2: WMGRISGSNGVADY (47-60)
ABR H3: RDRRLDYYENSAYFLGHDGFDI (98-119)

BLAST Sequence aligment against SARS-CoV-2

ABR H1:
>ORF1ab polyprotein [Severe acute respiratory syndrome coronavirus 2]
Sequence ID: UFC29138.1 Length: 7096
Range 1: 2351 to 2356

Score:19.7 bits(39), Expect:16334,
Method:,
Identities:5/6(83%), Positives:5/6(83%), Gaps:0/6(0%)

```
Query  3     FTNFAV  8
             F NFAV
Sbjct  2351  FSNFAV  2356
```

Range 2: 4811 to 4817

Score:17.2 bits(33), Expect:139818,
Method:,
Identities:5/7(71%), Positives:6/7(85%), Gaps:0/7(0%)

```
Query  3     FTNFAVS  9
             F +FAVS
Sbjct  4811  FYDFAVS  4817
```

ABR H2

>surface glycoprotein, partial [Severe acute respiratory syndrome coronavirus 2]
Sequence ID: QRX29628.1 Length: 682
Range 1: 357 to 365

Score:24.0 bits(49), Expect:1281,
Method:,
Identities:8/11(73%), Positives:8/11(72%), Gaps:2/11(18%)

```
Query  4    RISGSNGVADY  14
            RIS  N VADY
Sbjct  357  RIS--NXVADY  365
```

ABR H3

>ORF1a polyprotein [Severe acute respiratory syndrome coronavirus 2]
Sequence ID: QWC82475.1 Length: 4405
Range 1: 1893 to 1907

```
Score:26.5 bits(55), Expect:522,
Method:,
Identities:10/16(63%), Positives:11/16(68%), Gaps:4/16(25%)

Query  2     DRRLD-YY--ENSAYF  14
             DR LD YY  +NS YF
Sbjct  1893  DRKLDNYYKKDNS-YF  1907
```

A0A7T0PXI8

>tr|A0A7T0PXI8|A0A7T0PXI8_HUMAN Immunglobulin heavy chain variable region (Fragment) OS=Homo sapiens OX=9606 PE=2 SV=1
EVQLVESGGGVVRPGGSLRLSCAASGFSFDDYAMTWVRQAPGKGLEWVSGITYNGGSTGY
ADSVKGRFTISRDNAKNSLYLQMTNLRAGDTAVYFCARDATYCGGDCYLDYWGQGALVTVSS

BLAST Sequence aligment against nr

Query: tr|A0A7T0PXI8|A0A7T0PXI8_HUMAN Immunglobulin heavy chain variable region (Fragment) OS=Homo sapiens OX=9606 PE=2 SV=1 Query ID: lcl|Query_52619 Length: 122

>anti-SARS-CoV-2 immunoglobulin heavy chain variable region, partial [Homo sapiens]
Sequence ID: QKY76690.1 Length: 125
Range 1: 1 to 125

Score:197 bits(502), Expect:8e-63,
Method:Compositional matrix adjust.,
Identities:98/125(78%), Positives:107/125(85%), Gaps:3/125(2%)

```
Query  1    EVQLVESGGGVVRPGGSLRLSCAASGFSFDDYAMTWVRQAPGKGLEWVSGITYNGGSTGY  60
            EVQLVESGGG+V+PG SLRLSCAASGF+FDDYAM WVRQAPGKGLEWVSGI++N GS GY
Sbjct  1    EVQLVESGGGLVQPGRSLRLSCAASGFTFDDYAMHWVRQAPGKGLEWVSGISWNSGSIGY  60

Query  61   ADSVKGRFTISRDNAKNSLYLQMTNLRAGDTAVYFCARDATYCGG---DCYLDYWGQGAL  117
            ADSVKGRFTISRDNAKNSLYLQM +LRA DT +Y+CA+D  Y  G     Y DYWGQG L
Sbjct  61   ADSVKGRFTISRDNAKNSLYLQMNSLRAEDTGLYYCAKDINYDSGGYHKNYFDYWGQGTL  120

Query  118  VTVSS  122
            VTVSS
Sbjct  121  VTVSS  125
```

Paratome analysis

paratome_1_seq_6561_122_bp
ABR H1: FSFDDYAMT (27-35)
ABR H2: WVSGITYNGGSTGY (47-60)
ABR H3: RDATYCGGDCYLDY (98-111)

BLAST Sequence aligment against SARS-CoV-2

ABR H1: FSFDDYAMT (27-35)
>ORF1ab polyprotein [Severe acute respiratory syndrome coronavirus 2]
Sequence ID: QYV18445.1 Length: 7096
Range 1: 1087 to 1090

Score:18.0 bits(35), Expect:68513,
Method:,
Identities:4/4(100%), Positives:4/4(100%), Gaps:0/4(0%)

```
Query  3     FDDY  6
             FDDY
Sbjct  1087  FDDY  1090
```

ABR H2: WVSGITYNGGSTGY (47-60)
>ORF1ab polyprotein [Severe acute respiratory syndrome coronavirus 2]
Sequence ID: UKT45678.1 Length: 7093
Range 1: 4731 to 4735

Score:18.0 bits(35), Expect:179260,
Method:,
Identities:5/5(100%), Positives:5/5(100%), Gaps:0/5(0%)

```
Query  10    GSTGY  14
             GSTGY
Sbjct  4731  GSTGY  4735

ABR H3: RDATYCGGDCYLDY (98-111)
>ORF1a polyprotein, partial [Severe acute respiratory syndrome coronavirus
2]
Sequence ID: QRX11300.1 Length: 3727
Range 1: 187 to 196

Score:21.0 bits(42), Expect:15118,
Method:,
Identities:6/10(60%), Positives:7/10(70%), Gaps:0/10(0%)
Query  2    DATYCGGDCY  11
            D  +CG DCY
Sbjct  187  DNNFCGPDCY  196
```

A0A5C2G586

>tr|A0A5C2G586|A0A5C2G586_HUMAN IGH c149_heavy__IGHV3-20_IGHD3-10_IGHJ6 (Fragment) OS=Homo sapiens OX=9606 PE=2 SV=1
EVQLVESGGSVVRPGGSLRLSCAASGFTFDDYAMTWVRQAPGKGLEWVCGINWNGGSTGY
ADSVKGRFTISRDNAKNSLYLQMNSLRADDTALYYCARNGALYYPGSEWPTLPLKYYYGMDVWGQGTTVTVSS

BLAST Sequence aligment against nr

Query: tr|A0A5C2G586|A0A5C2G586_HUMAN IGH c149_heavy__IGHV3-20_IGHD3-10_IGHJ6 (Fragment) OS=Homo sapiens OX=9606 PE=2 SV=1 Query ID: lcl|Query_19769 Length: 133

>anti-SARS-CoV-2 spike protein immunoglobulin heavy chain variable region, partial [Homo sapiens]
Sequence ID: QKY76519.1 Length: 129
>anti-SARS-CoV-2 spike protein immunoglobulin heavy chain variable region, partial [Homo sapiens]
Sequence ID: QKY76525.1 Length: 129
>anti-SARS-CoV-2 spike protein immunoglobulin heavy chain variable region, partial [Homo sapiens]
Sequence ID: QKY76528.1 Length: 129
Range 1: 1 to 129

Score:209 bits(531), Expect:4e-67,
Method:Compositional matrix adjust.,
Identities:109/133(82%), Positives:114/133(85%), Gaps:4/133(3%)

```
Query  1    EVQLVESGGSVVRPGGSLRLSCAASGFTFDDYAMTWVRQAPGKGLEWVCGINWNGGSTGY  60
            EVQLVESGG VVRPGGSLRLSCAASGFTFDDY M+WVRQAPGKGLEWV  INWNGGSTGY
Sbjct  1    EVQLVESGGGVVRPGGSLRLSCAASGFTFDDYGMSWVRQAPGKGLEWVSAINWNGGSTGY  60

Query  61   ADSVKGRFTISRDNAKNSLYLQMNSLRADDTALYYCARNGALYYPGSEWPTLPLKYYYGM  120
            ADSVKGRFTISRDNAKNSLYLQMNSLRA+DTALY+CAR  +     S W      YYY M
Sbjct  61   ADSVKGRFTISRDNAKNSLYLQMNSLRAEDTALYHCARRRSSSRYSSGW----YMYYYYM  116

Query  121  DVWGQGTTVTVSS  133
            DVWG+GTTVTVSS
Sbjct  117  DVWGKGTTVTVSS  129
```

Paratome analysis

paratome_1_seq_6688_133_bp
ABR H1: FTFDDYAMT (27-35)
ABR H2: WVCGINWNGGSTGY (47-60)
ABR H3: RNGALYYPGSEWPTLPLKYYYGMDV (98-122)

BLAST Sequence aligment against SARS-CoV-2

ABR H1: FTFDDYAMT (27-35)
>ORF1ab polyprotein [Severe acute respiratory syndrome coronavirus 2]
Sequence ID: QYV18445.1 Length: 7096
Range 1: 1087 to 1090

Score:18.0 bits(35), Expect:68513,
Method:,
Identities:4/4(100%), Positives:4/4(100%), Gaps:0/4(0%)

```
Query  3     FDDY  6
             FDDY
Sbjct  1087  FDDY  1090
```

>ORF3a protein [Severe acute respiratory syndrome coronavirus 2]
Sequence ID: QZU11999.1 Length: 275

```
Range 1: 207 to 212

Score:18.5 bits(36), Expect:48026,
Method:,
Identities:5/6(83%), Positives:5/6(83%), Gaps:0/6(0%)

Query  1    FTFDDY  6
            FT DDY
Sbjct  207  FTSDDY  212
```

```
ABR H2: WVCGINWNGGSTGY (47-60)
>ORF1ab polyprotein [Severe acute respiratory syndrome coronavirus 2]
Sequence ID: UHE81962.1 Length: 7096
Range 1: 4402 to 4406

Score:19.3 bits(38), Expect:62036,
Method:,
Identities:4/5(80%), Positives:5/5(100%), Gaps:0/5(0%)

Query  1     WVCGI  5
             WVCG+
Sbjct  4402  WVCGV  4406

>membrane glycoprotein [Severe acute respiratory syndrome coronavirus 2]
Sequence ID: UKO22082.1 Length: 222
Range 1: 73 to 79

Score:19.7 bits(39), Expect:43115,
Method:,
Identities:6/7(86%), Positives:6/7(85%), Gaps:1/7(14%)

Query  5   INWN-GG  10
           INWN GG
Sbjct  73  INWNTGG  79
```

```
ABR H3: RNGALYYPGSEWPTLPLKYYYGMDV (98-122)
>ORF1ab polyprotein [Severe acute respiratory syndrome coronavirus 2]
Sequence ID: QKM77286.1 Length: 7096
Range 1: 5620 to 5634

Score:23.1 bits(47), Expect:10988,
Method:,
Identities:9/21(43%), Positives:9/21(42%), Gaps:9/21(42%)

Query  4     ALYYPGSEWPTLPLK---YYY  21
             ALYYP         K   YYY
Sbjct  5620  ALYYPS------XXKEYXYYY  5634
```

A0A7S5EUT4

>tr|A0A7S5EUT4|A0A7S5EUT4_HUMAN IGH c1335_heavy_IGHV3-11_IGHD4-23_IGHJ4 (Fragment) OS=Homo sapiens OX=9606 PE=2 SV=1
QVQLVESGGGLVKPGGSLRLSCAASGLTFSDYFMSWVRQAPGKGLEWLSYINNRGGHIYY
ADSVKGRFTISRDNAQNSLYLQMNSLRAEDTAVYYCASVPTFDGNFRRPLYYFDSWGQGT
LVTVSS

BLAST Sequence aligment against nr

Query: tr|A0A7S5EUT4|A0A7S5EUT4_HUMAN IGH c1335_heavy_IGHV3-11_IGHD4-23_IGHJ4 (Fragment) OS=Homo sapiens OX=9606 PE=2 SV=1 Query ID: lcl|Query_51035 Length: 126

>anti-SARS-CoV-2 spike protein immunoglobulin heavy chain variable region, partial [Homo sapiens]
Sequence ID: QKY76453.1 Length: 120
Range 1: 1 to 120

Score:202 bits(513), Expect:1e-64,
Method:Compositional matrix adjust.,
Identities:101/126(80%), Positives:107/126(84%), Gaps:6/126(4%)

```
Query  1    QVQLVESGGGLVKPGGSLRLSCAASGLTFSDYFMSWVRQAPGKGLEWLSYINNRGGHIYY  60
            QVQLVESGGGLVKPGGSLRLSCAASG TFSDY+MSW+RQAPGKGLEW+SYI++ G  IYY
Sbjct  1    QVQLVESGGGLVKPGGSLRLSCAASGFTFSDYYMSWIRQAPGKGLEWVSYISSSGITIYY  60

Query  61   ADSVKGRFTISRDNAQNSLYLQMNSLRAEDTAVYYCASVPTFDGNFRRPLYYFDSWGQGT  120
            ADSVKGRFTISRDNA+NSLYLQMNSLRAEDTAVYYC  V         P  YF  WGQGT
Sbjct  61   ADSVKGRFTISRDNAKNSLYLQMNSLRAEDTAVYYCTGV------VAAPAEYFQHWGQGT  114

Query  121  LVTVSS  126
            LVTVSS
Sbjct  115  LVTVSS  120
```

>anti SARS-CoV-2 immunoglobulin heavy chain, partial [Homo sapiens]
Sequence ID: QNT09657.1 Length: 129
Range 1: 1 to 129

Score:201 bits(512), Expect:3e-64,
Method:Compositional matrix adjust.,
Identities:102/129(79%), Positives:111/129(86%), Gaps:3/129(2%)

```
Query  1    QVQLVESGGGLVKPGGSLRLSCAASGLTFSDYFMSWVRQAPGKGLEWLSYINNRGGHIYY  60
            QVQLVESGGGLVKPGGSLRLSCAASG TFSDY+M+W+RQAPGKGLEW+SYI++ G  IYY
Sbjct  1    QVQLVESGGGLVKPGGSLRLSCAASGFTFSDYYMTWIRQAPGKGLEWVSYISSSGSTIYY  60

Query  61   ADSVKGRFTISRDNAQNSLYLQMNSLRAEDTAVYYCASVPTFDGNFR---RPLYYFDSWG  117
            ADSVKGRFTISRDNA+NSLYLQMNSLRAEDTAVYYCA      G +R   R   +FD WG
Sbjct  61   ADSVKGRFTISRDNAKNSLYLQMNSLRAEDTAVYYCARARGSSGWYRIGTRWGNWFDPWG  120

Query  118  QGTLVTVSS  126
            QGTLVTVSS
Sbjct  121  QGTLVTVSS  129
```

Paratome analysis

paratome_1_seq_22986_126_bp
ABR H1: LTFSDYFMS (27-35)
ABR H2: WLSYINNRGGHIYYA (47-61)
ABR H3: ASVPTFDGNFRRPLYYFDS (97-115)

BLAST Sequence aligment against SARS-CoV-2

ABR H1: LTFSDYFMS (27-35)

>ORF1ab polyprotein [Severe acute respiratory syndrome coronavirus 2]
Sequence ID: UHX88987.1 Length: 7096
Range 1: 5547 to 5550

Score:19.3 bits(38), Expect:23321,
Method:,
Identities:4/4(100%), Positives:4/4(100%), Gaps:0/4(0%)

```
Query  5     DYFM  8
             DYFM
Sbjct  5547  DYFM  5550
```

Range 2: 4468 to 4472

Score:17.6 bits(34), Expect:97565,
Method:,
Identities:4/5(80%), Positives:5/5(100%), Gaps:0/5(0%)

```
Query  2     TFSDY  6
             TFS+Y
Sbjct  4468  TFSNY  4472
```

ABR H2: WLSYINNRGGHIYYA (47-61)

Query: unnamed protein product Query ID: lcl|Query_15311 Length: 15

>ORF1a polyprotein, partial [Severe acute respiratory syndrome coronavirus 2]
Sequence ID: UCW21460.1 Length: 4288
Range 1: 15 to 19

Score:19.7 bits(39), Expect:50869,
Method:,
Identities:5/5(100%), Positives:5/5(100%), Gaps:0/5(0%)

```
Query  9   GGHIY  13
           GGHIY
Sbjct  15  GGHIY  19
```

>nucleocapsid phosphoprotein, partial [Severe acute respiratory syndrome coronavirus 2]
Sequence ID: UDJ53547.1 Length: 266
Range 1: 177 to 181

Score:19.3 bits(38), Expect:71485,
Method:,
Identities:4/5(80%), Positives:4/5(80%), Gaps:0/5(0%)

```
Query  1    WLSYI  5
            WL YI
Sbjct  177  WLTYI  181
```

ABR H3: ASVPTFDGNFRRPLYYFDS (97-115)

Query: unnamed protein product Query ID: lcl|Query_52520 Length: 19

>ORF1ab polyprotein [Severe acute respiratory syndrome coronavirus 2]
Sequence ID: UJR43981.1 Length: 7092
Range 1: 5169 to 5176

Score:21.4 bits(43), Expect:22123,

```
Method:,
Identities:6/8(75%), Positives:6/8(75%), Gaps:0/8(0%)

Query  9     NFRRPLYY  16
             NFR  LYY
Sbjct  5169  NFRSVLYY  5176

>surface glycoprotein [Severe acute respiratory syndrome coronavirus 2]
Sequence ID: UBE13906.1 Length: 1273
Range 1: 26 to 38

Score:19.3 bits(38), Expect:123128,
Method:,
Identities:6/13(46%), Positives:7/13(53%), Gaps:0/13(0%)

Query  4   PTFDGNFRRPLYY  16
           P +   FRR  YY
Sbjct  26  PAYTNSFRRGVYY  38
```

A0A5C2FU04

>tr|A0A5C2FU04|A0A5C2FU04_HUMAN IGL c441_light_IGKV3D-15_IGKJ1 (Fragment) OS=Homo sapiens OX=9606 PE=2 SV=1
EILMTQSPATLSVSPGERVTLSCWASQSISSYLAWYQQKPGQAPRLLFYGASTRATGIPA
RFSASGSGTEFTLTISSLQSEDFAVYYCQQYNNWPRAFGQGTTVEIK

BLAST Sequence aligment against nr

Query: tr|A0A5C2FU04|A0A5C2FU04_HUMAN IGL c441_light_IGKV3D-15_IGKJ1 (Fragment) OS=Homo sapiens OX=9606 PE=2 SV=1 Query ID: lcl|Query_4889
Length: 107

>anti-SARS-CoV-2 spike protein immunoglobulin light chain variable region, partial [Homo sapiens]
Sequence ID: QKY76193.1 Length: 107
>anti-SARS-CoV-2 spike protein immunoglobulin light chain variable region, partial [Homo sapiens]
Sequence ID: QKY76252.1 Length: 107
Range 1: 1 to 107

Score:197 bits(501), Expect:3e-63,
Method:Compositional matrix adjust.,
Identities:97/107(91%), Positives:99/107(92%), Gaps:0/107(0%)

```
Query  1    EILMTQSPATLSVSPGERVTLSCWASQSISSYLAWYQQKPGQAPRLLFYGASTRATGIPA  60
            EI+MTQSPATLSVSPGER TLSC ASQS+SS LAWYQQKPGQAPRLL YGASTRATGIPA
Sbjct  1    EIVMTQSPATLSVSPGERATLSCRASQSVSSNLAWYQQKPGQAPRLLIYGASTRATGIPA  60

Query  61   RFSASGSGTEFTLTISSLQSEDFAVYYCQQYNNWPRAFGQGTTVEIK  107
            RFS SGSGTEFTLTISSLQSEDFAVYYCQQYNNWP  FGQGT VEIK
Sbjct  61   RFSGSGSGTEFTLTISSLQSEDFAVYYCQQYNNWPGTFGQGTKVEIK  107
```

Paratome analysis

paratome_1_seq_6875_107_bp
ABR L1: QSISSYLA (27-34)
ABR L2: LLFYGASTRAT (46-56)
ABR L3: QQYNNWPRA (89-97)

BLAST Sequence aligment against SARS-CoV-2

ABR L1: QSISSYLA (27-34)
>ORF1ab polyprotein [Severe acute respiratory syndrome coronavirus 2]
Sequence ID: UFC70978.1 Length: 7091
Range 1: 1021 to 1027

Score:18.9 bits(37), Expect:26492,
Method:,
Identities:5/7(71%), Positives:5/7(71%), Gaps:0/7(0%)

```
Query  1     QSISSYL  7
             Q IS YL
Sbjct  1021  QTISGYL  1027
```

>surface glycoprotein [Severe acute respiratory syndrome coronavirus 2]
Sequence ID: QZI78246.1 Length: 1271
Range 1: 688 to 693

Score:19.3 bits(38), Expect:18584,
Method:,
Identities:5/6(83%), Positives:5/6(83%), Gaps:0/6(0%)

```
Query  1    QSISSY  6
            QSIS Y
Sbjct  688  QSISAY  693
```

ABR L2: LLFYGASTRAT (46-56)
>ORF1ab polyprotein [Severe acute respiratory syndrome coronavirus 2]
Sequence ID: QSO13661.1 Length: 7096
Range 1: 4763 to 4768

Score:18.9 bits(37), Expect:51342,
Method:,
Identities:5/6(83%), Positives:5/6(83%), Gaps:0/6(0%)

```
Query  1     LLFYGA  6
             LLFY A
Sbjct  4763  LLFYAA  4768
```

ABR L3: QQYNNWPRA (89-97)

>surface glycoprotein [Severe acute respiratory syndrome coronavirus 2]
Sequence ID: UFD05785.1 Length: 1271
Range 1: 1205 to 1211

Score:21.4 bits(43), Expect:3979,
Method:,
Identities:5/7(71%), Positives:6/7(85%), Gaps:0/7(0%)

```
Query  1     QQYNNWP  7
             +QY NWP
Sbjct  1205  EQYINWP  1211
```

A0A5C2GH36

>tr|A0A5C2GH36|A0A5C2GH36_HUMAN IG c401_light_IGKV3-20_IGKJ4 (Fragment)
OS=Homo sapiens OX=9606 PE=2 SV=1
EIVLMQSPGTLSLSPGERATLSCRPSQSVSSNHLAWYQQKPGQAPRLLIYGASVRATGIP
DRFSGSGSGTDFTLTISRLEPEDFAVYYCHQYGRSPTFGGGTRVEIK

BLAST Sequence aligment against nr

Query: tr|A0A5C2GH36|A0A5C2GH36_HUMAN IG c401_light_IGKV3-20_IGKJ4
(Fragment) OS=Homo sapiens OX=9606 PE=2 SV=1 Query ID: lcl|Query_18782
Length: 107

>anti-SARS-CoV-2 spike protein immunoglobulin light chain variable region,
partial [Homo sapiens]
Sequence ID: QKY76187.1 Length: 108
>anti-SARS-CoV-2 spike protein immunoglobulin light chain variable region,
partial [Homo sapiens]
Sequence ID: QKY76198.1 Length: 108
Range 1: 1 to 108

Score:198 bits(503), Expect:1e-63,
Method:Compositional matrix adjust.,
Identities:100/108(93%), Positives:101/108(93%), Gaps:1/108(0%)

```
Query  1    EIVLMQSPGTLSLSPGERATLSCRPSQSVSSNHLAWYQQKPGQAPRLLIYGASVRATGIP  60
            EIVL QSPGTLSLSPGERATLSCR SQSVSSN LAWYQQKPGQAPRLLIYGAS RATGIP
Sbjct  1    EIVLTQSPGTLSLSPGERATLSCRASQSVSSNFLAWYQQKPGQAPRLLIYGASSRATGIP  60

Query  61   DRFSGSGSGTDFTLTISRLEPEDFAVYYCHQYGRSP-TFGGGTRVEIK  107
            DRFSGSGSGTDFTLTISRLEPEDFAVYYC QYGRSP TFG GTR+EIK
Sbjct  61   DRFSGSGSGTDFTLTISRLEPEDFAVYYCQQYGRSPITFGQGTRLEIK  108
```

Paratome analysis

paratome_1_seq_6953_107_bp
ABR L1: QSVSSNHLA (27-35)
ABR L2: LLIYGASVRAT (47-57)
ABR L3: HQYGRSP (90-96)

BLAST Sequence aligment against SARS-CoV-2

ABR L1: QSVSSNHLA (27-35)
>ORF1ab polyprotein [Severe acute respiratory syndrome coronavirus 2]
Sequence ID: QNN88284.1 Length: 7096
Range 1: 3739 to 3744

Score:18.5 bits(36), Expect:47890,
Method:,
Identities:5/6(83%), Positives:5/6(83%), Gaps:0/6(0%)

```
Query  2     SVSSNH  7
             SV SNH
Sbjct  3739  SVTSNH  3744
```

ABR L2: LLIYGASVRAT (47-57)
>surface glycoprotein [Severe acute respiratory syndrome coronavirus 2]
Sequence ID: UDB79764.1 Length: 1273
Range 1: 1012 to 1020

Score:17.6 bits(34), Expect:150151,
Method:,
Identities:5/9(56%), Positives:6/9(66%), Gaps:0/9(0%)

```
Query  2     LIYGASVRA  10
             LI  A +RA
Sbjct  1012  LIXXAXIRA  1020

ABR L3: HQYGRSP (90-96)
>ORF1ab polyprotein [Severe acute respiratory syndrome coronavirus 2]
Sequence ID: UGC79169.1 Length: 7108
Range 1: 96 to 100

Score:19.3 bits(38), Expect:14481,
Method:,
Identities:5/5(100%), Positives:5/5(100%), Gaps:0/5(0%)

Query  2   QYGRS  6
           QYGRS
Sbjct  96  QYGRS  100
```

A0A5C2GPU5

>tr|A0A5C2GPU5|A0A5C2GPU5_HUMAN IG c893_heavy_IGHV3-15_IGHD3-16_IGHJ4 (Fragment) OS=Homo sapiens OX=9606 PE=2 SV=1
EVQLVESGGGLVKPGGSLRLSCAASGFSFSNAWMSWVRQAPGKGLEWVGHIKSKADGGTT
DYAVPVKPIFTISRDDSKNTLYLQLNSLKTEDTAMYYCTTGGGALSYFDYWGQGTLVTVSS

BLAST Sequence aligment against nr

Query: tr|A0A5C2GPU5|A0A5C2GPU5_HUMAN IG c893_heavy_IGHV3-15_IGHD3-16_IGHJ4 (Fragment) OS=Homo sapiens OX=9606 PE=2 SV=1 Query ID: lcl|Query_90911
Length: 121

>cross-reactive anti-SARS-CoV-2 immunoglobulin heavy chain variable region, partial [Homo sapiens]
Sequence ID: QRG26521.1 Length: 127
Range 1: 1 to 127

Score:206 bits(524), Expect:3e-66,
Method:Compositional matrix adjust.,
Identities:105/127(83%), Positives:112/127(88%), Gaps:6/127(4%)

```
Query  1    EVQLVESGGGLVKPGGSLRLSCAASGFSFSNAWMSWVRQAPGKGLEWVGHIKSKADGGTT  60
            +VQLVESGGGLVKPGGSLRLSCAASGF+FSNAWMSWVRQAPGKGLEWVG IKSK DGGTT
Sbjct  1    QVQLVESGGGLVKPGGSLRLSCAASGFTFSNAWMSWVRQAPGKGLEWVGRIKSKTDGGTT  60

Query  61   DYAVPVKPIFTISRDDSKNTLYLQLNSLKTEDTAMYYCTT---GGGALSYF---DYWGQG  114
            DYA PVK  FTISRDDSKNTLYLQ+NSLKTEDTA+YYCTT   G G+L Y+   D WG+G
Sbjct  61   DYAAPVKGRFTISRDDSKNTLYLQMNSLKTEDTAVYYCTTEEPGAGSLYYYYYMDVWGKG  120

Query  115  TLVTVSS  121
            T VTVSS
Sbjct  121  TTVTVSS  127
```

>anti-SARS-CoV-2 immunoglobulin gamma heavy chain variable region, partial [Homo sapiens]
Sequence ID: QJU69707.1 Length: 127
Range 1: 1 to 127

Score:213 bits(541), Expect:1e-68,
Method:Compositional matrix adjust.,
Identities:107/127(84%), Positives:111/127(87%), Gaps:6/127(4%)

```
Query  1    EVQLVESGGGLVKPGGSLRLSCAASGFSFSNAWMSWVRQAPGKGLEWVGHIKSKADGGTT  60
            EVQLVESGGGLVKPGGSLRLSCAASGF+FSNAWMSWVRQAPGKGLEWVG IKSK DGGTT
Sbjct  1    EVQLVESGGGLVKPGGSLRLSCAASGFTFSNAWMSWVRQAPGKGLEWVGRIKSKTDGGTT  60

Query  61   DYAVPVKPIFTISRDDSKNTLYLQLNSLKTEDTAMYYCTTGG------GALSYFDYWGQG  114
            DYA PVK  FTISRDDSKNTLYLQ+NSLKTEDTA+YYCTT        G+  Y DYWGQG
Sbjct  61   DYAAPVKGRFTISRDDSKNTLYLQMNSLKTEDTAVYYCTTDRVYDYIWGSYRYLDYWGQG  120

Query  115  TLVTVSS  121
            TLVTVSS
Sbjct  121  TLVTVSS  127
```

Paratome analysis

paratome_1_seq_7036_121_bp
ABR H1: FSFSNAWMS (27-35)
ABR H2: WVGHIKSKADGGTTDY (47-62)
ABR H3: TGGGALSYFDY (100-110)

BLAST Sequence aligment against SARS-CoV-2

ABR H1: FSFSNAWMS (27-35)

```
No significant similarity found.

ABR H2: WVGHIKSKADGGTTDY (47-62)
>ORF1a polyprotein [Severe acute respiratory syndrome coronavirus 2]
Sequence ID: UKR34990.1 Length: 4401
Range 1: 1301 to 1311

Score:19.3 bits(38), Expect:84318,
Method:,
Identities:7/11(64%), Positives:8/11(72%), Gaps:0/11(0%)

Query  5     IKSKADGGTTD  15
             I  K DGGTT+
Sbjct  1301  IPTKKDGGTTE  1311
ABR H3: TGGGALSYFDY (100-110)

>ORF1ab polyprotein [Severe acute respiratory syndrome coronavirus 2]
Sequence ID: UDG47217.1 Length: 7096
Range 1: 4838 to 4847

Score:21.0 bits(42), Expect:8671,
Method:,
Identities:6/10(60%), Positives:7/10(70%), Gaps:0/10(0%)

Query  2     GGGALSYFDY  11
             G  A SY+DY
Sbjct  4838  GNAAISYYDY  4847

>ORF3a protein [Severe acute respiratory syndrome coronavirus 2]
Sequence ID: UHP61412.1 Length: 275
Range 1: 204 to 211

Score:19.7 bits(39), Expect:25152,
Method:,
Identities:6/8(75%), Positives:6/8(75%), Gaps:2/8(25%)

Query  6    LSYF--DY  11
            LSYF  DY
Sbjct  204  LSYFTSDY  211
```

A0A5C2H2C4

>tr|A0A5C2H2C4|A0A5C2H2C4_HUMAN IG c1116_light_IGKV3-20_IGKJ2 (Fragment) OS=Homo sapiens OX=9606 PE=2 SV=1
EIVLTQSPGTLSLSPGDRATLSCRASRSVSSAQLTWYQQRPGQAPRLLLYATSTRATGVP
DRFSGSGSGTDFTLTISKVQPEDFAVYFCHQYESSPRTFGQGTKLEIK

BLAST Sequence aligment against nr

Query: tr|A0A5C2H2C4|A0A5C2H2C4_HUMAN IG c1116_light_IGKV3-20_IGKJ2 (Fragment) OS=Homo sapiens OX=9606 PE=2 SV=1 Query ID: lcl|Query_10487
Length: 108

>anti-SARS-CoV-2 spike protein immunoglobulin light chain variable region, partial [Homo sapiens]
Sequence ID: QKY76271.1 Length: 108
Range 1: 1 to 108

Score:188 bits(478), Expect:1e-59,
Method:Compositional matrix adjust.,
Identities:89/108(82%), Positives:101/108(93%), Gaps:0/108(0%)

```
Query  1    EIVLTQSPGTLSLSPGDRATLSCRASRSVSSAQLTWYQQRPGQAPRLLLYATSTRATGVP  60
            EIVLTQSPGTLSLSPG+RATLSCRAS+SVSS  L WYQQ+PGQAPRLL++  S+RATG+P
Sbjct  1    EIVLTQSPGTLSLSPGERATLSCRASQSVSSTFLAWYQQKPGQAPRLLIFGASSRATGIP  60

Query  61   DRFSGSGSGTDFTLTISKVQPEDFAVYFCHQYESSPRTFGQGTKLEIK  108
            DRFSGSGSGTDFTLTIS+++PEDFAVY+CHQY +SP TFGQGTKLEIK
Sbjct  61   DRFSGSGSGTDFTLTISRLEPEDFAVYYCHQYGTSPYTFGQGTKLEIK  108
```

Paratome analysis

paratome_1_seq_7482_108_bp
ABR L1: RSVSSAQLT (27-35)
ABR L2: LLLYATSTRAT (47-57)
ABR L3: HQYESSPR (90-97)

BLAST Sequence aligment against SARS-CoV-2

ABR L1: RSVSSAQLT (27-35)
>ORF1ab polyprotein [Severe acute respiratory syndrome coronavirus 2]
Sequence ID: UBU25355.1 Length: 7096
Range 1: 4404 to 4412

Score:18.9 bits(37), Expect:33499,
Method:,
Identities:6/9(67%), Positives:6/9(66%), Gaps:0/9(0%)

```
Query  1     RSVSSAQLT  9
             R VS A LT
Sbjct  4404  RGVSAARLT  4412
```

ABR L2: LLLYATSTRAT (47-57)

>ORF1a polyprotein [Severe acute respiratory syndrome coronavirus 2]
Sequence ID: QQO94433.1 Length: 4405
Range 1: 2176 to 2186

Score:19.7 bits(39), Expect:24963,
Method:,
Identities:6/11(55%), Positives:6/11(54%), Gaps:0/11(0%)

```
Query  1     LLLYATSTRAT  11
             LL   T TR T
Sbjct  2176  LLXXXTXTRST  2186
```

ABR L3: HQYESSPR (90-97)
No significant similarity found.

A0A7S5EWS1

>tr|A0A7S5EWS1|A0A7S5EWS1_HUMAN IGH c768_heavy_IGHV3-48_IGHD4-17_IGHJ3 (Fragment) OS=Homo sapiens OX=9606 PE=2 SV=1
EVQLEESGGGLVQPGGSLRLSCEVSGFTFRNYEMNWVRQAPGKGLEWVAYIGSFSSPTHY
AGSVRGRFTISRDNAKNSLYLQMNSLRADDTALYYCARTRKSDYGDYSEDEGMDVWGRGT
MVTVSS

BLAST Sequence aligment against nr

Query: tr|A0A7S5EWS1|A0A7S5EWS1_HUMAN IGH c768_heavy_IGHV3-48_IGHD4-17_IGHJ3 (Fragment) OS=Homo sapiens OX=9606 PE=2 SV=1 Query ID: lcl|Query_71519 Length: 126

>anti-SARS-CoV-2 spike protein immunoglobulin heavy chain variable region, partial [Homo sapiens]
Sequence ID: QKY76471.1 Length: 126
>anti-SARS-CoV-2 spike protein immunoglobulin heavy chain variable region, partial [Homo sapiens]
Sequence ID: QKY76492.1 Length: 126
Range 1: 1 to 126

Score:192 bits(488), Expect:1e-60,
Method:Compositional matrix adjust.,
Identities:94/126(75%), Positives:104/126(82%), Gaps:0/126(0%)

```
Query  1    EVQLEESGGGLVQPGGSLRLSCEVSGFTFRNYEMNWVRQAPGKGLEWVAYIGSFSSPTHY  60
            EVQL ESGGGLVQPGGSLRLSC  SGFTF +YEMNWVRQAPGKGLEWV+YI S  S  +Y
Sbjct  1    EVQLVESGGGLVQPGGSLRLSCAASGFTFSSYEMNWVRQAPGKGLEWVSYISSSGSAIYY  60

Query  61   AGSVRGRFTISRDNAKNSLYLQMNSLRADDTALYYCARTRKSDYGDYSEDEGMDVWGRGT  120
            A SV+GRFTISRDNAKNSLYLQMNSLR +DTA+YYCAR  +S Y D+      D WG+GT
Sbjct  61   ADSVKGRFTISRDNAKNSLYLQMNSLRVEDTAVYYCAREARSRYFDWLPSYYFDYWGQGT  120

Query  121  MVTVSS  126
            +VTVSS
Sbjct  121  LVTVSS  126
```

Paratome analysis

paratome_1_seq_7559_126_bp
ABR H1: FTFRNYEMN (27-35)
ABR H2: WVAYIGSFSSPTHY (47-60)
ABR H3: RTRKSDYGDYSEDEGMDV (98-115)

BLAST Sequence aligment against SARS-CoV-2

ABR H1: FTFRNYEMN (27-35)

>ORF1ab polyprotein [Severe acute respiratory syndrome coronavirus 2]
Sequence ID: UKG54270.1 Length: 7089
Range 1: 4461 to 4467

Score:21.4 bits(43), Expect:3973,
Method:,
Identities:5/7(71%), Positives:6/7(85%), Gaps:0/7(0%)

```
Query  2     TFRNYEM  8
             TF NY+M
Sbjct  4461  TFSNYQM  4467
```

ABR H2: WVAYIGSFSSPTHY (47-60)

>ORF1a polyprotein [Severe acute respiratory syndrome coronavirus 2]

Sequence ID: QWU48698.1 Length: 4402
Range 1: 2593 to 2600

Score:22.3 bits(45), Expect:5258,
Method:,
Identities:6/8(75%), Positives:7/8(87%), Gaps:0/8(0%)

```
Query  3     AYIGSFSS  10
             AY+G FSS
Sbjct  2593  AYVGTFSS  2600
```

ABR H3: RTRKSDYGDYSEDEGMDV (98-115)

>ORF1ab polyprotein, partial [Severe acute respiratory syndrome coronavirus 2]
Sequence ID: UFZ21166.1 Length: 7070
Range 1: 5500 to 5505

Score:22.3 bits(45), Expect:9684,
Method:,
Identities:6/6(100%), Positives:6/6(100%), Gaps:0/6(0%)

```
Query  4     KSDYGD  9
             KSDYGD
Sbjct  5500  KSDYGD  5505
```

Part 2. Analysis of immunoglobulin proteins overrepresented in infected cohorts when compared to PCR- individuals.

Analytical workflow

1. Select immunoglobulin protein sequences overrepresented with highest PCR+/- Log fold-change relative intensity exclusively in each infected asymptomatic, nonsevere and severe cohorts.
2. Protein BLAST sequence alignment against non-redundant protein database(nr)using compositional matrix adjustment (https://blast.ncbi.nlm.nih.gov/Blast.cgi?PROGRAM=blastp&PAGE_TYPE=BlastSearch&LINK_LOC=blasthome).
3. Identification of immunoglobulin sequences aligned with known function. Criteria: Score > 160 bits, Identity > 60%.
4. Identification of correlates of identified proteins with protective or disease associated capacity.

Input data:

Protein ID	Severe
A0A5C2GIT4	0.977
A0A5C2GDW3	0.795
A0A7S5C115	0.731

Protein ID	Nonsevere
A0A5C2G7I4	0.343
A0A5C2GPZ0	0.328
A0A7S5EYL7	0.296

Protein ID	Asymptomatic
A0A5C2GJF4	1.240
A0A5C2FZ03	0.793
A0A5C2G410	0.730

Severe patients

A0A5C2GIT4

BLAST Sequence aligment against nr

>tr|A0A5C2GIT4|A0A5C2GIT4_HUMAN IG c17_heavy_IGHV5-51_IGHD5-12_IGHJ6 (Fragment) OS=Homo sapiens OX=9606 PE=2 SV=1
EVQLVQSGAEMKKPGESLRISCRGSGYTFTKYWIGWVRLMPGRGLEWMGIIFPRDSETRY
SPSFQGQVTISADKSIRTAYLQWTSLNVSDSATYYCARAKGIEAPGHYYGMDVWGHGTTV
TVSS

>anti-peanut 2S albumin immunoglobulin heavy chain variable region, partial [Homo sapiens]
Sequence ID: QVG74243.1 Length: 124
Range 1: 1 to 124

Score:196 bits(497), Expect:4e-62,
Method:Compositional matrix adjust.,
Identities:93/124(75%), Positives:105/124(84%), Gaps:0/124(0%)

```
Query  1    EVQLVQSGAEMKKPGESLRISCRGSGYTFTKYWIGWVRLMPGRGLEWMGIIFPRDSETRY  60
            EVQLVQSGAE+KKPGESL+ISC GSG+ FT YWI WVR MPG+GLEWMG I+P D++T Y
Sbjct  1    EVQLVQSGAEVKKPGESLKISCEGSGFIFTNYWIAWVRQMPGKGLEWMGSIYPADADTTY  60

Query  61   SPSFQGQVTISADKSIRTAYLQWTSLNVSDSATYYCARAKGIEAPGHYYGMDVWGHGTTV  120
            SPSFQGQVTISADKSI TAYLQW+SL  SD+A+YYCAR +      +YYGMDVWG GTTV
Sbjct  61   SPSFQGQVTISADKSINTAYLQWSSLKASDTASYYCARRVPGNSYYYYGMDVWGQGTTV  120

Query  121  TVSS  124
            TVSS
Sbjct  121  TVSS  124
```

Correlate: Risk of allergy

A0A5C2GDW3

>tr|A0A5C2GDW3|A0A5C2GDW3_HUMAN IGH + IGL c109_heavy_IGHV3-11_IGHD3-10_IGHJ4 (Fragment) OS=Homo sapiens OX=9606 PE=2 SV=1
QVQLVESGGGLVKPGGSLRLSCAASGFTFSDYYMSWVRQAPGKGLEWISYIRNRGNGMYY
ADSVKGRFTISRDNAKNSLYLQMSSLQADDTAVYYCARISGFYGSEVFDYWGQGTLLTVS
S

BLAST Sequence aligment against nr

>anti-peanut 2S albumin immunoglobulin heavy chain variable region, partial [Homo sapiens]
Sequence ID: QVG74146.1 Length: 122
Range 1: 1 to 122

Score:202 bits(514), Expect:1e-64,
Method:Compositional matrix adjust.,
Identities:101/122(83%), Positives:110/122(90%), Gaps:1/122(0%)

```
Query  1    QVQLVESGGGLVKPGGSLRLSCAASGFTFSDYYMSWVRQAPGKGLEWISYIRNRGNGMYY  60
            QVQLVESGGGLVKPGGSLRLSCAASGFTFSDYYMSW+RQAPGKGLEW+SYI + G+ +YY
Sbjct  1    QVQLVESGGGLVKPGGSLRLSCAASGFTFSDYYMSWIRQAPGKGLEWVSYISSSGSTIYY  60

Query  61   ADSVKGRFTISRDNAKNSLYLQMSSLQADDTAVYYCAR-ISGFYGSEVFDYWGQGTLLTV  119
            ADSVKGRFTISRDNAKNSLYLQM+SL+A+DTAVYYCAR      G   FDYWGQGTL+TV
Sbjct  61   ADSVKGRFTISRDNAKNSLYLQMNSLRAEDTAVYYCARDYYDSSGYYYFDYWGQGTLVTV  120
```

```
Query  120  SS  121
            SS
Sbjct  121  SS  122
```

Correlate: Risk of allergy

A0A7S5C115

>tr|A0A7S5C115|A0A7S5C115_HUMAN IGH c1867_heavy_IGHV3-49_IGHD2-2_IGHJ6 (Fragment) OS=Homo sapiens OX=9606 PE=2 SV=1
EVQLVESGGGLVQPGRSLRLSCTGSGFTFGDFAMSWVRQAPGKGLEWVSLIRSNAYGGTT
EHAASVKGRFTISRDNSKSIAYLQMDSLQTEDTAVYYCTRGLPAIIGYYMDVWAKGTTVT
VSS

BLAST Sequence aligment against nr

>anti-GPIIb/IIIa immunoglobulin heavy chain variable region, partial [Homo sapiens]
Sequence ID: AAK77548.1 Length: 122
Range 1: 1 to 122

Score:206 bits(525), Expect:2e-66,
Method:Compositional matrix adjust.,
Identities:102/122(84%), Positives:108/122(88%), Gaps:0/122(0%)

```
Query  1    EVQLVESGGGLVQPGRSLRLSCTGSGFTFGDFAMSWVRQAPGKGLEWVSLIRSNAYGGTT  60
            EVQLVESGGGLVQPGRSLRLSCT SGFTFGD+AMSWVRQAPGKGLEWV  IRS AYGGTT
Sbjct  1    EVQLVESGGGLVQPGRSLRLSCTASGFTFGDYAMSWVRQAPGKGLEWVGFIRSKAYGGTT  60

Query  61   EHAASVKGRFTISRDNSKSIAYLQMDSLQTEDTAVYYCTRGLPAIIGYYMDVWAKGTTVT  120
            E+AASVKGRFTISRD+SKSIAYLQM+SL+TEDTAVYYCT   P    Y MDVW +GT VT
Sbjct  61   EYAASVKGRFTISRDDSKSIAYLQMNSLKTEDTAVYYCTVRSPGYYYYGMDVWGQGTLVT  120

Query  121  VS  122
            VS
Sbjct  121  VS  122
```

Correlate: Chronic idiopathic thrombocytopenic purpura (ITP) caused by an antibody reactive with platelet-associated antigens

>anti-GPIIb/IIIa immunoglobulin heavy chain variable region, partial [Homo sapiens]
Sequence ID: AAK77548.1 Length: 122
Range 1: 1 to 122

Score:206 bits(525), Expect:2e-66,
Method:Compositional matrix adjust.,
Identities:102/122(84%), Positives:108/122(88%), Gaps:0/122(0%)

```
Query  1    EVQLVESGGGLVQPGRSLRLSCTGSGFTFGDFAMSWVRQAPGKGLEWVSLIRSNAYGGTT  60
            EVQLVESGGGLVQPGRSLRLSCT SGFTFGD+AMSWVRQAPGKGLEWV  IRS AYGGTT
Sbjct  1    EVQLVESGGGLVQPGRSLRLSCTASGFTFGDYAMSWVRQAPGKGLEWVGFIRSKAYGGTT  60

Query  61   EHAASVKGRFTISRDNSKSIAYLQMDSLQTEDTAVYYCTRGLPAIIGYYMDVWAKGTTVT  120
            E+AASVKGRFTISRD+SKSIAYLQM+SL+TEDTAVYYCT   P    Y MDVW +GT VT
Sbjct  61   EYAASVKGRFTISRDDSKSIAYLQMNSLKTEDTAVYYCTVRSPGYYYYGMDVWGQGTLVT  120

Query  121  VS  122
            VS
Sbjct  121  VS  122
```


Correlate: Autoantibodies anti-platelet GPIIb/IIIa

Nonsevere patients

A0A5C2G7I4

>tr|A0A5C2G7I4|A0A5C2G7I4_HUMAN IGH c572_heavy__IGHV3-21_IGHD5-18_IGHJ5 (Fragment) OS=Homo sapiens OX=9606 PE=2 SV=1
EVQLVESGGGLVKPGGSLKLSCAASGFPFSAYTMTWVRQAPGKGLEWVSIISGSDSYISY
VDSVKGRFTISRDNANNALYLQMNSLRAEDTAVYYCARVEGLDPIDRWGQGTPVTVSS

BLAST Sequence aligment against nr

>myosin-reactive immunoglobulin heavy chain variable region, partial [Homo sapiens]
Sequence ID: AAD56259.1 Length: 118
Range 1: 1 to 117

Score:171 bits(432), Expect:3e-52,
Method:Compositional matrix adjust.,
Identities:92/117(79%), Positives:100/117(85%), Gaps:0/117(0%)

```
Query  1    EVQLVESGGGLVKPGGSLKLSCAASGFPFSAYTMTWVRQAPGKGLEWvsiisgsdsyisy  60
            EVQLVESGGGLV+PGGSL+LSCAASGF FS+Y+M WVRQAPGKGLEWVS IS +   I Y
Sbjct  1    EVQLVESGGGLVQPGGSLRLSCAASGFTFSSYSMNWVRQAPGKGLEWVSYISSTIITIYY  60

Query  61   vdsvKGRFTISRDNANNALYLQMNSLRAEDTAVYYCARVEGLDPIDRWGQGTPVTVS  117
             DSVKGRFTISRDNA N+LYLQMNSLRAEDTAVYYCAR +  +  D WGQGT VTVS
Sbjct  61   ADSVKGRFTISRDNAKNSLYLQMNSLRAEDTAVYYCARGDSSEAFDIWGQGTMVTVS  117
```

>anti-SARS-CoV-2 immunoglobulin gamma heavy chain, partial [Homo sapiens]
Sequence ID: QUX33738.1 Length: 116
Range 1: 1 to 116

Score:169 bits(428), Expect:8e-52,
Method:Compositional matrix adjust.,
Identities:93/118(79%), Positives:102/118(86%), Gaps:2/118(1%)

```
Query  1    EVQLVESGGGLVKPGGSLKLSCAASGFPFSAYTMTWVRQAPGKGLEWvsiisgsdsyisy  60
            EVQL+ESGGGLV+PGGSL+LSCAASGF FS+YTM WVRQAPGKGLEWVS I+   S I Y
Sbjct  1    EVQLLESGGGLVQPGGSLRLSCAASGFTFSSYTMNWVRQAPGKGLEWVSYITSDSSTIYY  60

Query  61   vdsvKGRFTISRDNANNALYLQMNSLRAEDTAVYYCARVEGLDPIDRWGQGTPVTVSS  118
             DSVKGRFTISRDNA N+LYLQMNSLRAEDTAVYYCAR + +D  D WG+GT VTVSS
Sbjct  61   ADSVKGRFTISRDNAKNSLYLQMNSLRAEDTAVYYCARNKAMD--DYWGRGTLVTVSS  116
```

Correlate: Protective capacity against SARS-CoV-2 and autoantibodies-mediated risk of myasthenia gravis

A0A5C2GPZ0

>tr|A0A5C2GPZ0|A0A5C2GPZ0_HUMAN IG c650_heavy_IGHV3-23_IGHD4-11_IGHJ4 (Fragment) OS=Homo sapiens OX=9606 PE=2 SV=1
EVQLLDSGGGSVQPGGSLRLSCAASGFYFSNYAMNWVRQAPGKGLQWVARISGTGGDTFY
ADSVKGRFTISRDNSKNILSLQMDSLRDEDTAVYFCAKDRLSSPKVSEAYFDYWGPGTLV
TVSS

BLAST Sequence aligment against nr

>anti-SARS-CoV-2 immunoglobulin gamma heavy chain, partial [Homo sapiens]
Sequence ID: QUX33727.1 Length: 121
Range 1: 1 to 121

Score:199 bits(506), Expect:2e-63,

```
Method:Compositional matrix adjust.,
Identities:100/124(81%), Positives:109/124(87%), Gaps:3/124(2%)

Query  1    EVQLLDSGGGSVQPGGSLRLSCAASGFYFSNYAMNWVRQAPGKGLQWVARISGTGGDTFY  60
            EVQLL+SGGG VQPGGSLRLSCAASGF FS+YAM+WVRQAPGKGL+WV+ ISG+GG T+Y
Sbjct  1    EVQLLESGGGLVQPGGSLRLSCAASGFTFSSYAMSWVRQAPGKGLEWVSAISGSGGSTYY  60

Query  61   ADSVKGRFTISRDNSKNILSLQMDSLRDEDTAVYFCAKDRLSSPKVSEAYFDYWGPGTLV  120
            ADSVKGRFTISRDNSKN L LQM+SLR EDTAVY+CAKDR SS      YFDYWG GTLV
Sbjct  61   ADSVKGRFTISRDNSKNTLYLQMNSLRAEDTAVYYCAKDRSSSWHY---YFDYWGQGTLV  117

Query  121  TVSS  124
            TVSS
Sbjct  118  TVSS  121
```

```
>anti-peanut 2S albumin immunoglobulin heavy chain variable region, partial
[Homo sapiens]
Sequence ID: QVG74500.1 Length: 122
Range 1: 1 to 122

Score:197 bits(502), Expect:7e-63,
Method:Compositional matrix adjust.,
Identities:100/124(81%), Positives:110/124(88%), Gaps:2/124(1%)

Query  1    EVQLLDSGGGSVQPGGSLRLSCAASGFYFSNYAMNWVRQAPGKGLQWVARISGTGGDTFY  60
            EVQLL+SGGG VQPGGSLRLSCAASGF FS+YAM+WVRQAPGKGL+WV+ ISG+GG T+Y
Sbjct  1    EVQLLESGGGLVQPGGSLRLSCAASGFTFSSYAMSWVRQAPGKGLEWVSAISGSGGSTYY  60

Query  61   ADSVKGRFTISRDNSKNILSLQMDSLRDEDTAVYFCAKDRLSSPKVSEAYFDYWGPGTLV  120
            ADSVKGRFTISRDNSKN L LQM+SLR EDTAVY+CAKDR SS  +S   FDYWG GTLV
Sbjct  61   ADSVKGRFTISRDNSKNTLYLQMNSLRAEDTAVYYCAKDRDSSGYLS--LFDYWGQGTLV  118

Query  121  TVSS  124
            TVSS
Sbjct  119  TVSS  122
```

```
>anti-hepatitis B surface antigen immunoglobulin heavy chain variable
region, partial [Homo sapiens]
Sequence ID: AAL57837.1 Length: 121
Range 1: 1 to 121

Score:196 bits(499), Expect:2e-62,
Method:Compositional matrix adjust.,
Identities:96/124(77%), Positives:109/124(87%), Gaps:3/124(2%)

Query  1    EVQLLDSGGGSVQPGGSLRLSCAASGFYFSNYAMNWVRQAPGKGLQWVARISGTGGDTFY  60
            EVQL++SGGG VQPGGSLRLSCAASGF FS+YAM+WVRQAPGKGL+WV+ IS  GG T+Y
Sbjct  1    EVQLVESGGGLVQPGGSLRLSCAASGFTFSSYAMSWVRQAPGKGLEWVSGISARGGSTYY  60

Query  61   ADSVKGRFTISRDNSKNILSLQMDSLRDEDTAVYFCAKDRLSSPKVSEAYFDYWGPGTLV  120
            ADSVKGRFTISRDNSKN L LQM+SLR EDTAVY+CAKDR    +++ A+FDYWG GTLV
Sbjct  61   ADSVKGRFTISRDNSKNTLYLQMNSLRAEDTAVYYCAKDR---GRIAAAHFDYWGQGTLV  117

Query  121  TVSS  124
            TVSS
Sbjct  118  TVSS  121
```

```
>Diabody 305 complex with EpoR [Homo sapiens]
Sequence ID: 4Y5X_J Length: 136
Range 1: 8 to 131

Score:195 bits(496), Expect:9e-62,
Method:Compositional matrix adjust.,
Identities:95/124(77%), Positives:106/124(85%), Gaps:0/124(0%)

Query  1    EVQLLDSGGGSVQPGGSLRLSCAASGFYFSNYAMNWVRQAPGKGLQWVARISGTGGDTFY  60
            EVQLL+SGGG VQPGGSLRLSCAASGF FS+YAM+WVRQAPGKGL+WV+ ISG+GG T+Y
```

```
Sbjct  8    EVQLLESGGGLVQPGGSLRLSCAASGFTFSSYAMSWVRQAPGKGLEWVSAISGSGGSTYY  67

Query  61   ADSVKGRFTISRDNSKNILSLQMDSLRDEDTAVYFCAKDRLSSPKVSEAYFDYWGPGTLV  120
            ADSVKGRFTISRDNSKN L LQM+SLR EDTAVY+C KDR++       YFD WG GT V
Sbjct  68   ADSVKGRFTISRDNSKNTLYLQMNSLRAEDTAVYYCVKDRVAVAGKGSYYFDSWGRGTTV  127

Query  121  TVSS  124
            TVSS
Sbjct  128  TVSS  131
```

Correlate: Protective capacity against SARS-CoV-2 and Hepatitis B virus, risk of allergy and autoantibodies protein binding/immune system

A0A7S5EYL7

>tr|A0A7S5EYL7|A0A7S5EYL7_HUMAN IGH c4094_heavy_IGHV3-21_IGHD5-18_IGHJ4 (Fragment) OS=Homo sapiens OX=9606 PE=2 SV=1
EVHLVESGGGLVKPGGSLRLSCAASGFTFSSYSMNWVRQAPGKGLEWVSSISSSSRSIYY
ADSVKGRFTISRDNAKNSLYLQMNSLRAEDTAVYYCASPLSRGYSYIFDYWGQGTLVTVSS

BLAST Sequence aligment against nr

>anti-SARS-CoV-2 immunoglobulin heavy chain variable region, partial [Homo sapiens]
Sequence ID: QTX15853.1 Length: 125
Range 1: 1 to 125

Score:199 bits(507), Expect:1e-63,
Method:Compositional matrix adjust.,
Identities:107/125(86%), Positives:114/125(91%), Gaps:4/125(3%)

```
Query  1    EVHLVESGGGLVKPGGSLRLSCAASGFTFSSYSMNWVRQAPGKGLEWVssissssrsiYY  60
            EV LVESGGG+VKPGGSLRLSCAASGFTFS+YSMNWVRQAPGKGLEWVSSISSSS  I+Y
Sbjct  1    EVQLVESGGGVVKPGGSLRLSCAASGFTFSTYSMNWVRQAPGKGLEWVSSISSSSTDIHY  60

Query  61   ADSVKGRFTISRDNAKNSLYLQMNSLRAEDTAVYYCASPLSRGY----SYIFDYWGQGTL  116
            ADS+KGRFTISRDNAKNSLYLQMNSLRAEDTAVYYCA    RG+    +YIFD+WGQGTL
Sbjct  61   ADSMKGRFTISRDNAKNSLYLQMNSLRAEDTAVYYCARDFHRGWYDHSAYIFDFWGQGTL  120

Query  117  VTVSS  121
            VTVSS
Sbjct  121  VTVSS  125
```

>anti-SARS-CoV-2 immunoglobulin gamma, partial [Homo sapiens]
Sequence ID: QTI96680.1 Length: 126
Range 1: 1 to 126

Score:197 bits(502), Expect:8e-63,
Method:Compositional matrix adjust.,
Identities:107/126(85%), Positives:110/126(87%), Gaps:5/126(3%)

```
Query  1    EVHLVESGGGLVKPGGSLRLSCAASGFTFSSYSMNWVRQAPGKGLEWVssissssrsiYY  60
            EV LVESGGGLV+PGGSLRLSCAASGFTFSSY MNWVRQAPGKGLEWVS ISSS  +IYY
Sbjct  1    EVQLVESGGGLVQPGGSLRLSCAASGFTFSSYEMNWVRQAPGKGLEWVSYISSSGSTIYY  60

Query  61   ADSVKGRFTISRDNAKNSLYLQMNSLRAEDTAVYYCASPLS-----RGYSYIFDYWGQGT  115
            ADSVKGRFTISRDNAKNSLYLQMNSLRAEDTAVYYCA   S       Y+ IFDYWGQGT
Sbjct  61   ADSVKGRFTISRDNAKNSLYLQMNSLRAEDTAVYYCARDYSYCSSTSCYTSIFDYWGQGT  120

Query  116  LVTVSS  121
            LVTVSS
Sbjct  121  LVTVSS  126
```

>anti-peanut 2S albumin immunoglobulin heavy chain variable region, partial [Homo sapiens]

Sequence ID: QVG74488.1 Length: 120
Range 1: 1 to 120

Score:196 bits(499), Expect:2e-62,
Method:Compositional matrix adjust.,
Identities:106/121(88%), Positives:111/121(91%), Gaps:1/121(0%)

```
Query  1    EVHLVESGGGLVKPGGSLRLSCAASGFTFSSYSMNWVRQAPGKGLEWVssisssssrsiYY  60
            EV LVESGGGLV+PGGSLRLSCAASGFTFSSY MNWVRQAPGKGLEWVS ISSSS +IYY
Sbjct  1    EVQLVESGGGLVQPGGSLRLSCAASGFTFSSYCMNWVRQAPGKGLEWVSYISSSSNTIYY  60

Query  61   ADSVKGRFTISRDNAKNSLYLQMNSLRAEDTAVYYCASPLSRGYSYIFDYWGQGTLVTVSS  121
            ADSVKGRFTISRDNAKNSLYLQMNSLRAEDTAVY+CA   + G+ Y FDYWGQGTLVTVSS
Sbjct  61   ADSVKGRFTISRDNAKNSLYLQMNSLRAEDTAVYFCARDKTSGWWY-FDYWGQGTLVTVSS  120
```

Correlate: Protective capacity against SARS-CoV-2 and risk of allergy

Asymptomatic cases

A0A5C2GJF4

>tr|A0A5C2GJF4|A0A5C2GJF4_HUMAN IG c470_heavy_IGHV3-33_IGHD1-26_IGHJ4 (Fragment) OS=Homo sapiens OX=9606 PE=2 SV=1
QVQLVQSGGGVVQPGGSLRLSCAASGFTFSSYGIHWVRQAPGKGLDWVAFIRSDGSNKYY
ADSVRGRFIISRDNSKNTLYLQMNSLRTDDAAVYYCAKGGDEWDLWGAHFDYWGQGTLVT
VSS

BLAST Sequence aligment against nr

>anti-SARS-CoV-2 immunoglobulin heavy chain variable region, partial [Homo sapiens]
Sequence ID: UKB89119.1 Length: 123
Range 1: 19 to 123

Score:181 bits(459), Expect:3e-56,
Method:Compositional matrix adjust.,
Identities:88/105(84%), Positives:94/105(89%), Gaps:0/105(0%)

```
Query  19   RLSCAASGFTFSSYGIHWVRQAPGKGLDWVAFIRSDGSNKYYADSVRGRFIISRDNSKNT  78
            RLSCAASGFTFSSYG+HWVRQAPGKGL+WVA I  DGSNKYYADSV+GRF ISRDNSKNT
Sbjct  19   RLSCAASGFTFSSYGMHWVRQAPGKGLEWVAVISYDGSNKYYADSVKGRFTISRDNSKNT  78

Query  79   LYLQMNSLRTDDAAVYYCAKGGDEWDLWGAHFDYWGQGTLVTVSS  123
            LYLQMNSLR +D AVYYCAKGG  +D  G +FDYWGQGTLVTVSS
Sbjct  79   LYLQMNSLRAEDTAVYYCAKGGGWYDYKGYYFDYWGQGTLVTVSS  123
```

>anti-glycoprotein VI immunoglobulin heavy chain variable region antibody, partial [Homo sapiens]
Sequence ID: AAN15190.1 Length: 119
Range 1: 19 to 119

Score:178 bits(452), Expect:2e-55,
Method:Compositional matrix adjust.,
Identities:88/105(84%), Positives:92/105(87%), Gaps:4/105(3%)

```
Query  19   RLSCAASGFTFSSYGIHWVRQAPGKGLDWVAFIRSDGSNKYYADSVRGRFIISRDNSKNT  78
            RLSCAASGFTFSSYG+HWVRQAPGKGL+WVAFIR DGSNKYYADSV+GRF ISRDNSKNT
Sbjct  19   RLSCAASGFTFSSYGMHWVRQAPGKGLEWVAFIRYDGSNKYYADSVKGRFTISRDNSKNT  78

Query  79   LYLQMNSLRTDDAAVYYCAKGGDEWDLWGAHFDYWGQGTLVTVSS  123
            LYLQMNSLR +D AVYYCAKG        A FDYWGQGTLVTVSS
Sbjct  79   LYLQMNSLRAEDTAVYYCAKG----PRIAASFDYWGQGTLVTVSS  119
```

Correlate: Protective capacity against SARS-CoV-2 and thrombosis.

A0A5C2FZ03

>tr|A0A5C2FZ03|A0A5C2FZ03_HUMAN IGL c497_light_IGKV1D-17_IGKJ1 (Fragment) OS=Homo sapiens OX=9606 PE=2 SV=1
DIQMTQSPSAMSASVGDRVTITCRASQGISNYLAWFQQKPGKVPKRLIYAASNLQSGVPS
RFSGSGSGTEFTLTISSLQSEDFATYYCLQHNSYPRTFGQGTKVEIK

BLAST Sequence aligment against nr

anti-SARS-CoV-2 immunoglobulin light chain variable region, partial [Homo sapiens]
Sequence ID: QKK35737.1 Length: 107
>immunoglobulin light chain variable region, partial [Homo sapiens]
Sequence ID: QXE98237.1 Length: 107

Range 1: 1 to 107

Score:204 bits(519), Expect:6e-66,
Method:Compositional matrix adjust.,
Identities:98/107(92%), Positives:103/107(96%), Gaps:0/107(0%)

```
Query  1    DIQMTQSPSAMSASVGDRVTITCRASQGISNYLAWFQQKPGKVPKRLIYAASNLQSGVPS  60
            DIQ+TQSPSAMSASVGDRVTITCRASQGI++ LAWFQQKPGKVPKRLIYAASNLQ+GVPS
Sbjct  1    DIQLTQSPSAMSASVGDRVTITCRASQGINDNLAWFQQKPGKVPKRLIYAASNLQNGVPS  60

Query  61   RFSGSGSGTEFTLTISSLQSEDFATYYCLQHNSYPRTFGQGTKVEIK  107
            RFSGSGSGTEFTLTISSLQ EDFATYYCLQHNSYP TFG GTK+EIK
Sbjct  61   RFSGSGSGTEFTLTISSLQPEDFATYYCLQHNSYPLTFGGGTKLEIK  107
```

>anti-ZIKV immunoglobulin light chain variable region, partial [Homo sapiens]
Sequence ID: AOT82813.1 Length: 110
Range 1: 1 to 107

Score:202 bits(513), Expect:5e-65,
Method:Compositional matrix adjust.,
Identities:97/107(91%), Positives:101/107(94%), Gaps:0/107(0%)

```
Query  1    DIQMTQSPSAMSASVGDRVTITCRASQGISNYLAWFQQKPGKVPKRLIYAASNLQSGVPS  60
            DI MTQSPS++SASVGDRVTITCRASQGI N L W+QQKPGK PKRLIYAAS+LQSGVPS
Sbjct  1    DIVMTQSPSSLSASVGDRVTITCRASQGIRNDLGWYQQKPGKAPKRLIYAASSLQSGVPS  60

Query  61   RFSGSGSGTEFTLTISSLQSEDFATYYCLQHNSYPRTFGQGTKVEIK  107
            RFSGSGSGTEFTLTISSLQ EDFATYYCLQHNSYPRTFGQGTKVEIK
Sbjct  61   RFSGSGSGTEFTLTISSLQPEDFATYYCLQHNSYPRTFGQGTKVEIK  107
```

>anti HBs antibody light-chain Fab fragment, partial [Homo sapiens]
Sequence ID: BAB18259.1 Length: 214
Range 1: 1 to 107

Score:202 bits(514), Expect:1e-63,
Method:Compositional matrix adjust.,
Identities:95/107(89%), Positives:101/107(94%), Gaps:0/107(0%)

```
Query  1    DIQMTQSPSAMSASVGDRVTITCRASQGISNYLAWFQQKPGKVPKRLIYAASNLQSGVPS  60
            DI++TQSPSAM+ASVGDRVTITCRASQGI NYL WFQQKPGKVPKRLIYAAS+LQSGVPS
Sbjct  1    DIELTQSPSAMAASVGDRVTITCRASQGIGNYLVWFQQKPGKVPKRLIYAASSLQSGVPS  60

Query  61   RFSGSGSGTEFTLTISSLQSEDFATYYCLQHNSYPRTFGQGTKVEIK  107
            RFSGSGSGTEFTLTISSLQ EDFATYYCL HN+YP +FG GTKVEIK
Sbjct  61   RFSGSGSGTEFTLTISSLQPEDFATYYCLHHNNYPLSFGGGTKVEIK  107
```

Correlate: Protective capacity against SARS-CoV-2, Zika virus and Hepatitis B virus

A0A5C2G410

>tr|A0A5C2G410|A0A5C2G410_HUMAN IGL c4031_light_IGKV3-15_IGKJ1 (Fragment) OS=Homo sapiens OX=9606 PE=2 SV=1
EIVMTQSPATVSVYPGERATLSCRASQSVSTNLAWYQQKPGQAPRLLMYGASTRATDIPL
RFSGSGSGTEFTLTISSLQSEDFAVYYCQHYHNWPRTFGQGTKVESE

BLAST Sequence aligment against nr

>rotavirus-specific intestinal-homing antibody light chain variable region, partial [Homo sapiens]
Sequence ID: AAW67418.1 Length: 109
Range 1: 3 to 107

```
Score:201 bits(512), Expect:8e-65,
Method:Compositional matrix adjust.,
Identities:96/105(91%), Positives:101/105(96%), Gaps:0/105(0%)

Query  1    EIVMTQSPATVSVYPGERATLSCRASQSVSTNLAWYQQKPGQAPRLLMYGASTRATDIPL  60
            EIVMTQSPAT+SV PGERATLSCRASQSVS+NLAWYQQKPGQAPRLL+YGASTRAT IP
Sbjct  3    EIVMTQSPATLSVSPGERATLSCRASQSVSSNLAWYQQKPGQAPRLLIYGASTRATGIPA  62

Query  61   RFSGSGSGTEFTLTISSLQSEDFAVYYCQHYHNWPRTFGQGTKVE  105
            RFSGSGSGTEFTLTISSLQSEDFAVYYCQ Y+NWPRTFGQGTKV+
Sbjct  63   RFSGSGSGTEFTLTISSLQSEDFAVYYCQQYNNWPRTFGQGTKVD  107

>anti-SARS-CoV-2 spike protein immunoglobulin light chain variable region,
partial [Homo sapiens]
Sequence ID: QKY76147.1 Length: 107
Range 1: 1 to 105

Score:199 bits(506), Expect:5e-64,
Method:Compositional matrix adjust.,
Identities:95/105(90%), Positives:100/105(95%), Gaps:0/105(0%)

Query  1    EIVMTQSPATVSVYPGERATLSCRASQSVSTNLAWYQQKPGQAPRLLMYGASTRATDIPL  60
            EIVMTQSPAT+SV PGERATLSCRASQSVS+NLAWYQQKPGQAPRLL+YGASTRAT IP
Sbjct  1    EIVMTQSPATLSVSPGERATLSCRASQSVSSNLAWYQQKPGQAPRLLIYGASTRATGIPA  60

Query  61   RFSGSGSGTEFTLTISSLQSEDFAVYYCQHYHNWPRTFGQGTKVE  105
            RFSGSGSGTEFTLTISSLQSEDFAVYYCQ Y+NWPRTFGQG +VE
Sbjct  61   RFSGSGSGTEFTLTISSLQSEDFAVYYCQQYNNWPRTFGQGNRVE  105
```

Correlate: Protective capacity against SARS-CoV-2 and rotavirus
