# Supplementary material for: Correlates with Vaccine Protective Capacity and COVID-19 Disease Symptoms Identified by Serum Proteomics in Vaccinated Individuals
Source: Molecules. 2022 Sep 13;27(18):5933. doi: 10.3390/molecules27185933 (PMC9500703; doi:10.3390/molecules27185933)
Supplement: Supplementary file 1 [file molecules-27-05933-s001.zip › Data file S4.pdf]

**Data file S4.** Analysis of selected non-immunoglobulin proteins underrepresented and overrepresented in infected cohorts when compared to PCR- individuals.

Interactions between enriched GO/KEGG ontology clusters for proteins overrepresented in infected cohorts when compared to PCR- cases. (A) Enriched ontology clusters colored by cluster ID. (B) Enriched ontology clusters pied by protein counts across cohorts. Circle node size is proportional to the number of input proteins fall under that term, and its color represent its cluster identity. Terms with a similarity score > 0.3 are linked by an edge (the thickness of the edge represents the similarity score). The network is visualized with Cytoscape. (C) Circos plot of protein overlap analysis.

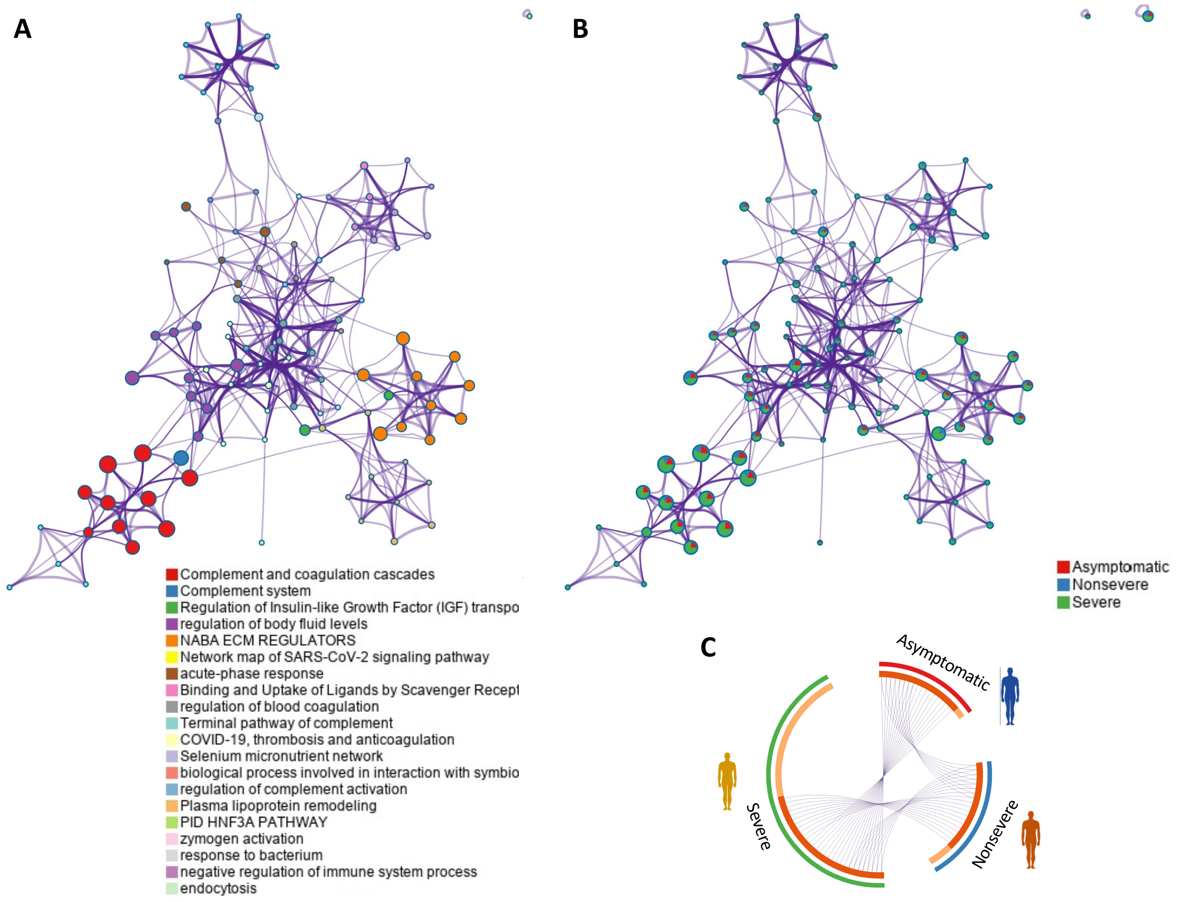

Interactions between enriched ontology clusters for proteins underrepresented in infected cohorts when compared to PCR- cases. (A) Enriched ontology clusters colored by cluster ID. (B) Enriched ontology clusters pied by protein counts across cohorts. Circle node size is proportional to the number of input proteins fall under that term, and its color represent its cluster identity. Terms with a similarity score > 0.3 are linked by an edge (the thickness of the edge represents the similarity score). The network is visualized with Cytoscape. (C) Circos plot of protein overlap analysis.

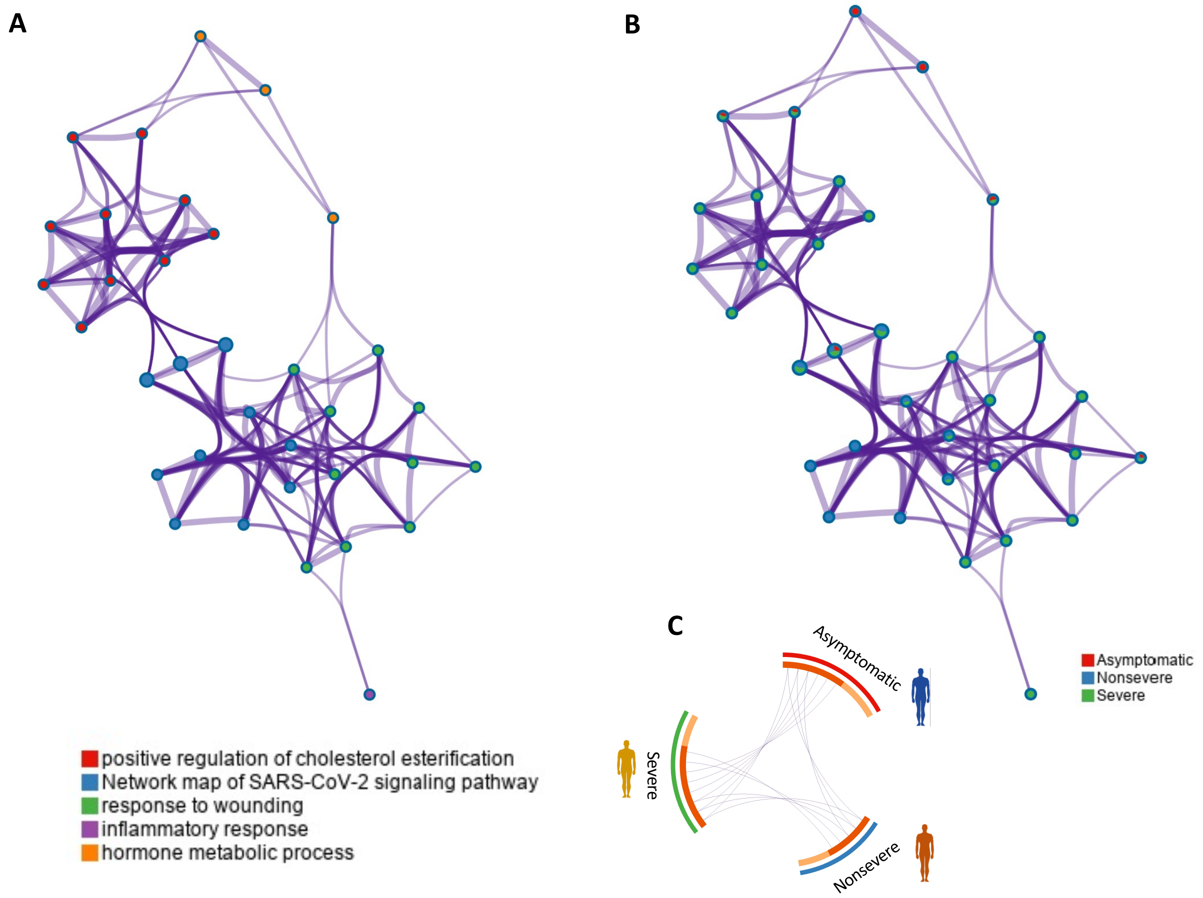

Characterization of differentially represented proteins in the biological process involved in interaction with symbiont (GO:0051702) using Reactome (https://reactome.org/) and QuickGO (https://www.ebi.ac.uk/QuickGO) algorithms.

**Activation in severe cohort.**

**WP15: Selenium (Se) micronutrient network.** Biochemical processes related to selenium in the context of metabolism, oxidation, immune response and inflammation. Role in protection against oxidative stress and proper cardiovascular function (Shimada et al., 2021) and ferroptotic cell death (Conrad et al., 2020). Deficiency in Selenium affects COVID-19 symptomatology (Taheri et al., 2021).
Shimada BK, Alfulaij N, Seale LA. The Impact of Selenium Deficiency on Cardiovascular Function. Int J Mol Sci. 2021 Oct 2;22(19):10713. doi: 10.3390/ijms221910713.
Conrad M, Proneth B. Selenium: Tracing Another Essential Element of Ferroptotic Cell Death. Cell Chem Biol. 2020 Apr 16;27(4):409-419. doi: 10.1016/j.chembiol.2020.03.012.
Taheri S, Asadi S, Nilashi M, Ali Abumalloh R, Ghabban NMA, Mohd Yusuf SY, Supriyanto E, Samad S. A literature review on beneficial role of vitamins and trace elements: Evidence from published clinical studies. J Trace Elem Med Biol. 2021 Sep;67:126789. doi: 10.1016/j.jtemb.2021.126789.

**R-HSA-166665: Terminal pathway of complement.** Hyperactivation of the complement and coagulation systems is recognized as part of the clinical syndrome of COVID-19.
Afzali B, Noris M, Lambrecht BN, Kemper C. The state of complement in COVID-19. *Nat Rev Immunol*. 2022;22(2):77-84. doi:10.1038/s41577-021-00665-1

**R-HSA-8963899: Plasma lipoprotein remodeling.** SARS-CoV-2 infection impacts on lipid metabolism. Decreased levels in HDL-C were reported in COVID-19 patients with reduction in HDL particles associated antioxidant, anti-inflammatory and anti-infectious properties.
Begue F, Tanaka S, Mouktadi Z, Rondeau P, Veeren B, Diotel N, Tran-Dinh A, Robert T, Vélia E, Mavingui P, Lagrange-Xélot M, Montravers P, Couret D, Meilhac O. Altered high-density lipoprotein composition and functions during severe COVID-19. Sci Rep. 2021 Jan 27;11(1):2291. doi: 10.1038/s41598-021-81638-1.

**GO:0031638: Zymogen activation.** Role of Zymogen in Proteolytic activation of the SARS-coronavirus spike protein.
Simmons G, Zmora P, Gierer S, Heurich A, Pöhlmann S. Proteolytic activation of the SARS-coronavirus spike protein: cutting enzymes at the cutting edge of antiviral research. Antiviral Res. 2013 Dec;100(3):605-14. doi: 10.1016/j.antiviral.2013.09.028.

**Activation in severe and nonsevere cohorts.**

**R-HAS-2173782: Binding and uptake of ligands by scavenger receptors.** Scavenger receptors bind free extracellular ligands as the initial step in clearance of the ligands from the body with a role in innate immunity and microbial pathogenesis (Areschoug and Gordon 2009).
Areschoug T, Gordon S. Scavenger receptors: role in innate immunity and microbial pathogenesis. Cell Microbiol. 2009 Aug;11(8):1160-9. doi: 10.1111/j.1462-5822.2009.01326.x.
high-density lipoprotein (HDL) scavenger receptor B type 1 (SR-B1) facilitates ACE2-dependent entry of SARS-CoV-2.
Wei C, Wan L, Yan Q, Wang X, Zhang J, Yang X, Zhang Y, Fan C, Li D, Deng Y, Sun J, Gong J, Yang X, Wang Y, Wang X, Li J, Yang H, Li H, Zhang Z, Wang R, Du P, Zong Y, Yin F, Zhang W, Wang N, Peng Y, Lin H, Feng J, Qin C, Chen W, Gao Q, Zhang R, Cao Y, Zhong H. HDL-scavenger receptor B type 1 facilitates SARS-CoV-2 entry. Nat Metab. 2020 Dec;2(12):1391-1400. doi: 10.1038/s42255-020-00324-0.

**Activation in severe and asymptomatic cohorts.**

M285: PID HNF3A pathway. R-HSA-4086398: Ca2+ pathway
to promote intracellular calcium release implicated in inflammatory response
Sugimura R, Li L. Noncanonical Wnt signaling in vertebrate development, stem cells, and diseases. Birth Defects Res C Embryo Today. 2010 Dec;90(4):243-56. doi: 10.1002/bdrc.20195.
Katoh M, Katoh M. WNT signaling pathway and stem cell signaling network. Clin Cancer Res. 2007 Jul 15;13(14):4042-5. doi: 10.1158/1078-0432.CCR-06-2316.

Cytokine profiles in COVID-19 patients are characterized increased levels of IL-1β, IL-2, IL-6, and TNF-α and increased NF-κB pathway activity. Recent evidence has shown that the upregulation of the WNT/β-catenin pathway is associated with inflammation, resulting in a cytokine storm in COVID-19 patients.
Vallée A, Lecarpentier Y, Vallée JN. Interplay of Opposing Effects of the WNT/β-Catenin Pathway and PPARγ and Implications for SARS-CoV2 Treatment. Front Immunol. 2021 Apr 13;12:666693. doi: 10.3389/fimmu.2021.666693.

**GO:0009617: Response to bacterium.** And to virus infection and symbiotic bacteria. Previously associated with COVID-19.

**GO:0030449: Regulation of complement activation.** Previously associated with COVID-19.

**WP4927: COVID-19 thrombosis and anticoagulation.** Previously associated with COVID-19.
https://www.wikipathways.org/index.php/Pathway:WP4927
Connors JM, Levy JH. COVID-19 and its implications for thrombosis and anticoagulation. Blood. 2020 Jun 4;135(23):2033-2040. doi: 10.1182/blood.2020006000.

**GO:0002683: Negative regulation of immune system process.** Previously associated with COVID-19.
Tahaghoghi-Hajghorbani S, Zafari P, Masoumi E, Rajabinejad M, Jafari-Shakib R, Hasani B, Rafiei A. The role of dysregulated immune responses in COVID-19 pathogenesis. Virus Res. 2020 Dec;290:198197. doi: 10.1016/j.virusres.2020.198197.

**Activation in all cohorts (highest in severe cases).**

**M3468: NABA ECM REGULATORS.**
**Extracellular matrix (ECM) proteins.**
Naba A, Clauser KR, Hoersch S, Liu H, Carr SA, Hynes RO. The matrisome: in silico definition and in vivo characterization by proteomics of normal and tumor extracellular matrices. Mol Cell Proteomics. 2012 Apr;11(4):M111.014647. doi: 10.1074/mcp.M111.014647.
Extracellular matrix changes damage the structure and function of COVID-19 lung tissue.
Leng L, Cao R, Ma J, Mou D, Zhu Y, Li W, Lv L, Gao D, Zhang S, Gong F, Zhao L, Qiu B, Xiang H, Hu Z, Feng Y, Dai Y, Zhao J, Wu Z, Li H, Zhong W. Pathological features of COVID-19-associated lung injury: a preliminary proteomics report based on clinical samples. Signal Transduct Target Ther. 2020 Oct 15;5(1):240. doi: 10.1038/s41392-020-00355-9.

**R-HAS-381426: Regulation if insulin-like growth factor (IGF) transport and uptake by insulin-like growth factor binding proteins (IGFBPs).** Higher IGF-1 concentrations are associated with a lower risk of COVID-19 mortality.
Fan X, Yin C, Wang J, Yang M, Ma H, Jin G, Song M, Hu Z, Shen H, Hang D. Pre-diagnostic circulating concentrations of insulin-like growth factor-1 and risk of COVID-19 mortality: results from UK Biobank. Eur J Epidemiol. 2021 Mar;36(3):311-318. doi: 10.1007/s10654-020-00709-1.

**GO:0030193: Regulation of blood coagulation.** Coagulopathy has been reported as associated with mortality in people with COVID-19 and is partially reflected by enhanced D-dimer levels.
Zadow EK, Wundersitz DWT, Hughes DL, Adams MJ, Kingsley MIC, Blacklock HA, Wu SSX, Benson AC, Dutheil F, Gordon BA. Coronavirus (COVID-19), Coagulation, and Exercise: Interactions That May Influence Health Outcomes. Semin Thromb Hemost. 2020 Oct;46(7):807-814. doi: 10.1055/s-0040-1715094.

**GO:0051702: Biological process involved in interaction with symbiont.**
http://www.informatics.jax.org/go/term/GO:0051702#myDataTable=results%3D500%26startIndex%3D0%26sort%3Dterm%26dir%3Dasc

**Export:** Text File | Excel File | MouseMine

| Symbol, Name | Chr | Annotated Term | Context | Proteoform | Inferred From | Reference(s) |
|---|---|---|---|---|---|---|
| Apoe, apolipoprotein E | 7 | positive regulation by host of viral process | | | P02649 | J:164563 |
| Cxcl1, chemokine (C-X-C motif) ligand 1 | 5 | positive regulation of neutrophil mediated killing of fungus | | | | J:213910 [PMID:10725737] |
| Gpx2, glutathione peroxidase 2 | 12 | biological process involved in interaction with symbiont | has the participant large intestine | | MGI:104887 \| MGI:106609 | J:103116 [PMID:12751789] |
| Hrg, histidine-rich glycoprotein | 16 | cytolysis by host of symbiont cells | | | P04196 | J:164563 |

| tr\|A0A0S2Z3D5\|A0A0S2Z3D5_HUMAN | Apolipoprotein E isoform 1 (Fragment) OS=Homo sapiens OX=9606 GN=APOE PE=2 SV=1 |
|---|---|

Log (Fold-change) Severe 0.157 UCI 0.081

| tr\|D3JV41\|D3JV41_HUMAN | C-X-C motif chemokine (Fragment) OS=Homo sapiens OX=9606 PE=3 SV=1 |
|---|---|

Log (Fold-change) Severe -0.123

| tr\|A0A087X1J7\|A0A087X1J7_HUMAN | Glutathione peroxidase OS=Homo sapiens OX=9606 GN=GPX3 PE=1 SV=1 |
|---|---|

Log (Fold-change) Severe 0.235 Nonsevere 0.127

| sp\|P04196\|HRG_HUMAN | Histidine-rich glycoprotein OS=Homo sapiens OX=9606 GN=HRG PE=1 SV=1 |
|---|---|

Log (Fold-change) Severe -0.382 Asymptomatic -0.218 Nonsevere -0.225 UCI -0.384

**Association with COVID**

**Apolipoprotein E isoform 1.**ApoE including ApoE1 expression is critical for assembly of infectious Hepatitis C virus in a strain-specific and cell type-dependent manner.
Weller R, Hueging K, Brown RJP, Todt D, Joecks S, Vondran FWR, Pietschmann T. Hepatitis C Virus Strain-Dependent Usage of Apolipoprotein E Modulates Assembly Efficiency and Specific Infectivity of Secreted Virions. J Virol. 2017 Aug 24;91(18):e00422-17. doi: 10.1128/JVI.00422-17.
COVID: higher risk associated with ApoE4 genetic variants. First report for ApoE1
Hubacek JA. Effects of selected inherited factors on susceptibility to SARS-CoV-2 infection and COVID-19 progression. Physiol Res. 2021 Dec 16;70(S2):S125-S134. doi: 10.33549/physiolres.934730.

**C-X-C motif chemokine.** C-X-C motif chemokines have been associated with COVID severity
Perreau M, Suffiotti M, Marques-Vidal P, Wiedemann A, Levy Y, Laouénan C, Ghosn J, Fenwick C, Comte D, Roger T, Regina J, Vollenweider P, Waeber G, Oddo M, Calandra T, Pantaleo G. The

cytokines HGF and CXCL13 predict the severity and the mortality in COVID-19 patients. Nat Commun. 2021 Aug 9;12(1):4888. doi: 10.1038/s41467-021-25191-5.
ID sequence is 100% identical to CXCL7_HUMAN - Platelet basic protein P02775.
Transcriptional analysis identified PPBP/CXCL7 as biomarker for disease severity and intubation.
Yatim N, Boussier J, Chocron R, Hadjadj J, Philippe A, Gendron N, Barnabei L, Charbit B, Szwebel TA, Carlier N, Pène F, Azoulay C, Khider L, Mirault T, Diehl JL, Guerin CL, Rieux-Laucat F, Duffy D, Kernéis S, Smadja DM, Terrier B. Platelet activation in critically ill COVID-19 patients. Ann Intensive Care. 2021 Jul 17;11(1):113. doi: 10.1186/s13613-021-00899-1.

**Glutathione peroxidase GPX3.** Essential micronutrients such as selenium (Se) and zinc (Zn) support anti-oxidative defense systems and are commonly depleted in severe disease. In COVID patients, Se supplementation significantly elevated Se, selenoprotein P levels and glutathione peroxidase 3 (GPx3) activity, with potential clinical significance for an adequate immune response in critically ill patients with severe COVID-19 acute respiratory distress syndrome (ARDS).
Notz Q, Herrmann J, Schlesinger T, Helmer P, Sudowe S, Sun Q, Hackler J, Roeder D, Lotz C, Meybohm P, Kranke P, Schomburg L, Stoppe C. Clinical Significance of Micronutrient Supplementation in Critically Ill COVID-19 Patients with Severe ARDS. Nutrients. 2021 Jun 20;13(6):2113. doi: 10.3390/nu13062113.

**Histidine-rich glycoprotein.** Higher Histidine-rich glycoprotein serum levels correlated with survival from severe COVID.
Völlmy F, van den Toorn H, Zenezini Chiozzi R, Zucchetti O, Papi A, Volta CA, Marracino L, Vieceli Dalla Sega F, Fortini F, Demichev V, Tober-Lau P, Campo G, Contoli M, Ralser M, Kurth F, Spadaro S, Rizzo P, Heck AJ. A serum proteome signature to predict mortality in severe COVID-19 patients. Life Sci Alliance. 2021 Jul 5;4(9):e202101099. doi: 10.26508/lsa.202101099.

Previously associated with COVID-19:
WP5115: Network map of SARS-CoV-2 signaling pathway
GO:0006953: Acute phase response
GO:0050878: regulation of body fluid levels
WP2806: Complement system
Hsa04610: Complement and coagulation cascades
GO:0006897: Endocytosis

**Suppression in severe cohort.**

**GO:0010873: Positive regulation of cholesterol esterification.** Cholesterol esterification is associated with liver function and mortality but this homeostatic mechanism may counteract the normally exacerbating effect of cholesterol on murine coronavirus mouse hepatitis virus (MHV) cytopathology.
Kaiser T, Kinny-Köster B, Bartels M, Berg T, Scholz M, Engelmann C, Seehofer D, Becker S, Ceglarek U, Thiery J. Cholesterol esterification in plasma as a biomarker for liver function and prediction of mortality. BMC Gastroenterol. 2017 Apr 20;17(1):57. doi: 10.1186/s12876-017-0614-9.
Cervin M, Anderson R. Modulation of coronavirus-mediated cell fusion by homeostatic control of cholesterol and fatty acid metabolism. J Med Virol. 1991 Oct;35(2):142-9. doi: 10.1002/jmv.1890350213.

Previously associated with COVID-19:
GO:0006954: Inflammatory response associated with negative regulation
WP5115: Network map of SARS-CoV-2 signaling pathway
GO:0009611: response to wounding
GO:0042445: hormone metabolic process
